# Time-resolved *in silico *modeling of fine-tuned cAMP signaling in platelets: feedback loops, titrated phosphorylations and pharmacological modulation

**DOI:** 10.1186/1752-0509-5-178

**Published:** 2011-10-28

**Authors:** Gaby Wangorsch, Elke Butt, Regina Mark, Katharina Hubertus, Jörg Geiger, Thomas Dandekar, Marcus Dittrich

**Affiliations:** 1 Department of Bioinformatics, Biocenter, University of Würzburg, Am Hubland, 97074 Würzburg, Germany; 2 Institute for Clinical Biochemistry & Pathobiochemistry, Grombühlstraße 12, 97080 Würzburg, Germany; 3 European Molecular Biology Laboratory (EMBL), Postfach 102209, 69012 Heidelberg, Germany

## Abstract

**Background:**

Hemostasis is a critical and active function of the blood mediated by platelets. Therefore, the prevention of pathological platelet aggregation is of great importance as well as of pharmaceutical and medical interest. Endogenous platelet inhibition is predominantly based on cyclic nucleotides (cAMP, cGMP) elevation and subsequent cyclic nucleotide-dependent protein kinase (PKA, PKG) activation. In turn, platelet phosphodiesterases (PDEs) and protein phosphatases counterbalance their activity. This main inhibitory pathway in human platelets is crucial for countervailing unwanted platelet activation. Consequently, the regulators of cyclic nucleotide signaling are of particular interest to pharmacology and therapeutics of atherothrombosis. Modeling of pharmacodynamics allows understanding this intricate signaling and supports the precise description of these pivotal targets for pharmacological modulation.

**Results:**

We modeled dynamically concentration-dependent responses of pathway effectors (inhibitors, activators, drug combinations) to cyclic nucleotide signaling as well as to downstream signaling events and verified resulting model predictions by experimental data. Experiments with various cAMP affecting compounds including anti-platelet drugs and their combinations revealed a high fidelity, fine-tuned cAMP signaling in platelets without cross-talk to the cGMP pathway. The model and the data provide evidence for two independent feedback loops: PKA, which is activated by elevated cAMP levels in the platelet, subsequently inhibits adenylyl cyclase (AC) but as well activates PDE3. By multi-experiment fitting, we established a comprehensive dynamic model with one predictive, optimized and validated set of parameters. Different pharmacological conditions (inhibition, activation, drug combinations, permanent and transient perturbations) are successfully tested and simulated, including statistical validation and sensitivity analysis. Downstream cyclic nucleotide signaling events target different phosphorylation sites for cAMP- and cGMP-dependent protein kinases (PKA, PKG) in the vasodilator-stimulated phosphoprotein (VASP). VASP phosphorylation as well as cAMP levels resulting from different drug strengths and combined stimulants were quantitatively modeled. These predictions were again experimentally validated. High sensitivity of the signaling pathway at low concentrations is involved in a fine-tuned balance as well as stable activation of this inhibitory cyclic nucleotide pathway.

**Conclusions:**

On the basis of experimental data, literature mining and database screening we established a dynamic *in silico *model of cyclic nucleotide signaling and probed its signaling sensitivity. Thoroughly validated, it successfully predicts drug combination effects on platelet function, including synergism, antagonism and regulatory loops.

## Background

Cyclic nucleotide signaling is the main inhibitory pathway in platelets *in vivo *that balances platelet activation. The complex regulation of this pathway includes endothelium released factors activating platelet nucleotide cyclases and in consequence cyclic nucleotide-dependent protein kinases that in turn phosphorylate major components of the platelet activation pathways thus preventing platelet aggregation [1]. Platelet phosphodiesterases (PDEs) counterbalance the action of nucleotide cyclases [2-5]. Regulators of cAMP levels are of strong pharmacological interest and have clinical and therapeutic implications [4].

Computational modeling can advance understanding of cellular signaling in thrombosis and hemostasis and elucidates furthermore the fine-tuned cAMP signaling in platelets. Great effort has been devoted to model endothelial layer maintenance and senescence [6] as well as thrombus development [7]. Here we model time-resolved cyclic nucleotide pathway signaling in human platelets involving specific parameters for metabolism, regulation and different effector strengths by means of a data-based *in silico *modeling approach.

Investigation of signaling pathways by kinetic modeling is commonly applied [8-11] and stimulates new biological insights into the modeled system. Combined with statistical validation based on experimental data, dynamic models can serve as a platform for testing hypotheses e.g. on pharmacological interventions on the system. Furthermore, the predictive features of kinetic models allow leading and directing biochemical experiments and elicit novel and unexpected biological findings.

**Figure 1:**
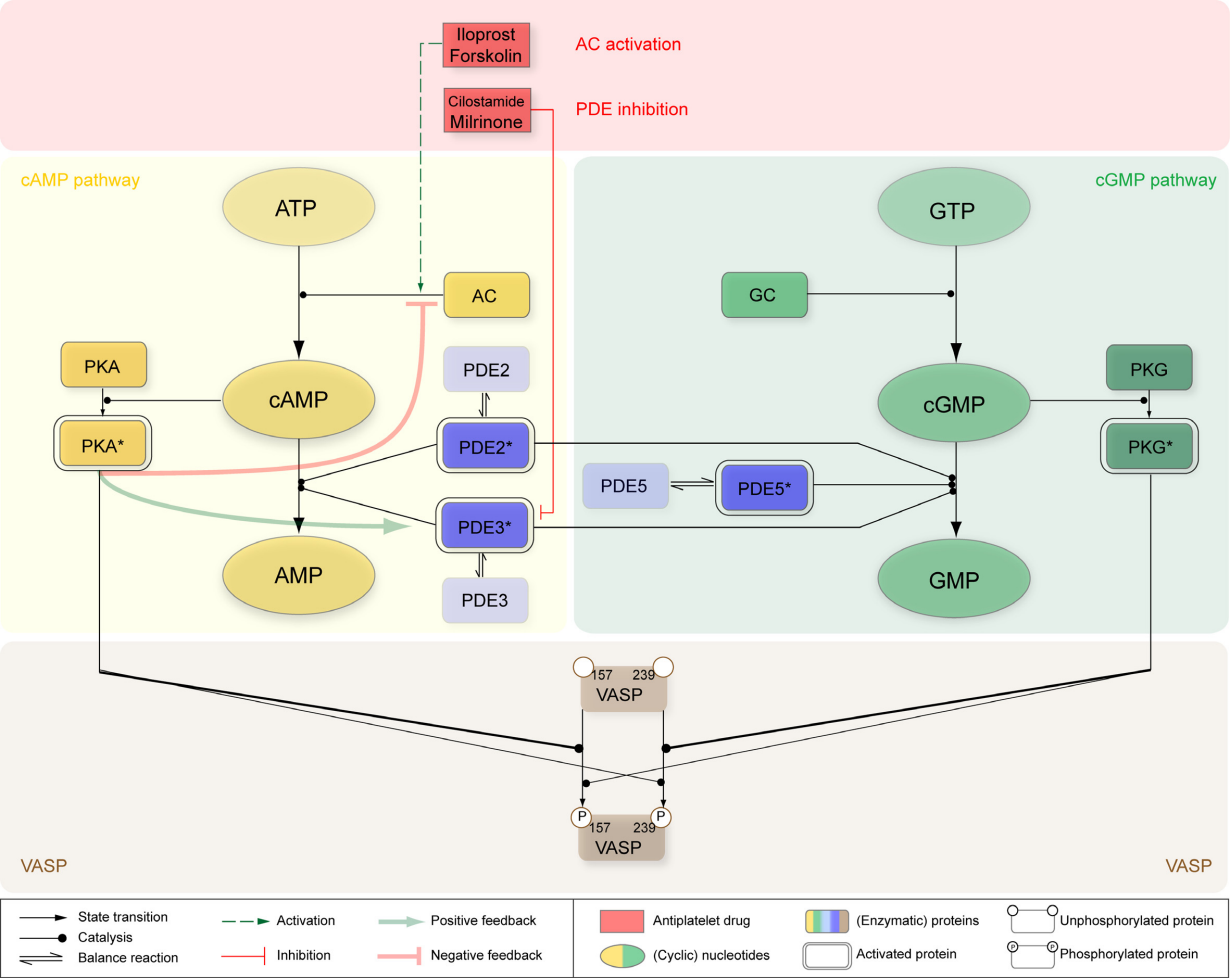
**Topological scheme of modeled cyclic nucleotide signaling pathways**. Main components of cyclic nucleotide signaling pathways and their cross-talk and downstream effects are depicted: Major signaling components of the simplified cAMP signaling pathway are shown in yellow, pivotal components of cGMP signaling in green and the three central cyclic nucleotide degrading phosphodiesterases (PDEs) are depicted in blue. Both, cAMP and cGMP signaling share in downstream effects on VASP (brown), which is phosphorylated at both, Ser157 and Ser239 differentially upon cyclic nucleotide dependent activation of their corresponding protein kinases (PKA, PKG). Levels of cAMP and cGMP react sensitively to pathway modulating compounds such as inhibitors of PDEs (Cilostamide, Milrinone) as well as adenylyl cyclase (AC) stimulating drugs (Iloprost, Forskolin) shown in red rectangles. This direct activation (green dashed arrow) and/or inhibition (red arrow) leads to an elevated cAMP level which subsequently feeds back on PDE3 (positive feedback) as well as on AC (negative feedback), as indicated by bold green and red colored arrows.

Here we present a first kinetic model on platelet signaling including time-resolved interplay and downstream effects of cyclic nucleotide signaling pathways (Figure 1).

In the following, our quantitative dynamic model, based on a system of ordinary differential equations (ODEs) is set up according to our philosophy (see below): Using a data-driven modeling approach, it enables us to correctly model the behavior of measured pathway components over time as well as to test the effects of drug combinations. It estimates individual effects of PDE-specific inhibitors and activators of adenylyl cyclase (AC) at different strengths and combinations and allows the prediction of feedback loops. We investigate cAMP accumulation after stimulation of adenylyl cyclase by Iloprost and Forskolin as well as the effects of inhibition of cAMP degrading PDEs by Cilostamide or Milrinone using individual concentration parameters for each platelet PDE isoform (PDE2, PDE3 and PDE5). The reliability of the model is tested and validated with experimental data of human platelets. It allows the prediction of effects of platelet inhibiting drugs and drug combinations on platelet cAMP pathway taking drug interactions, time-scales and regulatory loops into account.

## Results

### Modeling approach

Although the main players of inhibitory signal transduction in human platelets are well investigated, their interplay is still not fully understood. Since platelet inhibition and its up- and downstream signaling events influence this fine-tuned balance around platelet activation and quiescence, we here focus on a pharmacological motivated modeling approach, driven by data-based model validation.

**Figure 2:**
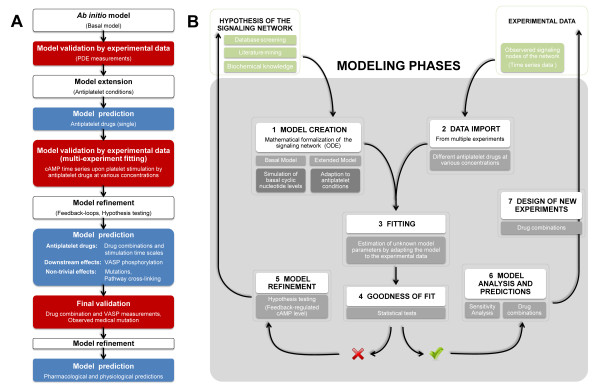
**Model development processes**. **(A) **Establishment of the network structure and straightforward model development. Within three main steps, the ***ab initio ***model is iteratively expanded and refined by means of experimental data and statistical (hypothesis) testing. Subsequent model predictions again serve for data-based model validation and statistical model selection. **(B) **Seven main modeling phases guided the way from the platelet signaling network creation and to the model-based design and prediction of new experiments within this study. These single modeling steps (white boxes) are further explained (gray boxes) referring to our study on platelet signaling.

Our modeling approach is as follows: To model the basal levels of platelet cAMP and cGMP we first established a dynamic *in silico *model based on a set of ordinary differential equations, comprising prior knowledge of specific kinetic constants and concentrations of the signaling components (see Appendix). We then performed experimental measurements of human platelets stimulating the system with various doses of PDE inhibitors and AC activators. All collected data were used to fit the model parameters (multi-experiment fitting), iteratively expanding and improving the ab initio model and its predictions by means of experimental data and statistical model testing (Figure 2A). This allowed us to differentiate between competing models (implementing different biological hypotheses, e.g. presence or absence of feedback regulation). Hypothesis testing and data-based model selection lead to the most parsimonious but reasonable model which was finally used to predict the effects for novel drug combinations as well as downstream-signaling events (VASP phosphorylation). These predictions again lead to the design of new experiments to validate the predictive model. The modeling and model validation itself was conducted within several modeling phases (Figure 2B), focusing on the goodness of fit regarding the model refinement processes.

### Set-up of a basal model of unstimulated platelets

**Figure 3:**
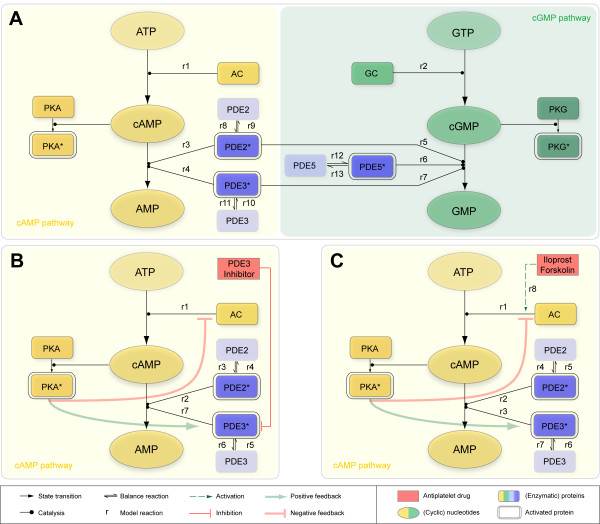
**Reaction scheme under basal and elevated cAMP conditions**. **(A) **Reaction scheme for defined basal levels of the cyclic nucleotides cAMP and cGMP hold in unstimulated platelets. A constant generation of cyclic nucleotides by the corresponding cyclase (reaction *r*1,*r*2) is balanced by degradation accomplished by active phosphodiesterases PDE2, PDE3 and the cGMP-specific PDE5 (reactions *r*8-*r*13) of the cyclic nucleotides (reactions *r*3-*r*7). The explicit formulation of the underlying reactions *r*1 to *r*13 is given in the Appendix. Reaction scheme for feedback-controlled regulation of cAMP levels considering cAMP-elevating treatment (PDE3 inhibition, AC activation) thereby forcing platelet inhibition (anti-platelet effect): **(B) **Platelet PDE3 inhibition (competitive inhibition via Milrinone, Cilostamide). The cAMP influx (reaction *r*1) and cAMP degradation (reaction *r*2,*r*7) via active phosphodiesterases PDE2, PDE3 (reactions *r*3-*r*6) act as a counterbalance. **(C) **Activation of AC via Iloprost and Forskolin. Elevated cAMP level due to the increased cyclase activity (reaction *r*8) is enzymatically degraded (reaction *r*2,*r*3) mediated by active platelet PDEs (reactions *r*4-r7).

We developed a dynamic model reflecting the major cyclic nucleotide pathway topology as illustrated in Figure 1 and specified in the Appendix. It integrates information about enzyme isoforms in platelets, gathered from a previously established proteome and transcriptome database of the human platelet [12,13] and literature mining: In the resting (non-stimulated) platelet, adenylyl and guanylyl cyclase (GC) and the three major phosphodiesterases PDE2, PDE3 and PDE5 together maintain basal cAMP and cGMP levels of 4 μM and 0.4 μM, respectively [14]. All known pivotal PDEs expressed in platelets were included: PDE2A (cGMP-stimulated), PDE3A (cGMP-inhibited) and PDE5A (cGMP-specific) and their respective enzymatic activity was modeled by Michaelis Menten kinetics as detailed in [2]. In particular, cAMP shows positively cooperative kinetic effects, resulting in a Hill coefficient of 2 with respect to PDE2 catalysis [15]. Moreover, PDE2 is modeled as a cGMP stimulated homodimer resulting in increased activity at physiological concentrations (1-10 μM), while V_max _remains unchanged [16]. Primarily, cAMP degradation is provided by PDE3 (80%), regulating basal cAMP levels [16]. Because of its cGMP specificity, PDE5 is included in the basal model but plays only a minor role under basal conditions and is insignificant for cAMP regulation. Under basal conditions (unstimulated platelet), we assumed an overall AC activity in platelets to 7 ± 2 μM/min according to data reported in literature [17-19]. This leads to the parsimonious but reasonable network structure of the resting state (Figure 3A).

### Model and experimental data indicate low effective PDE concentrations in resting human platelets

The central components of the platelet cyclic nucleotide pathways are the PDEs. Although, data on enzyme kinetics for many isoforms (including those expressed in the platelet) are available, no data on enzyme concentration in human platelets for any isoform are currently accessible. Therefore, we measured intracellular concentrations of the major phosphodiesterases in human platelets. After calibration with recombinant expressed PDEs by Western blot this yields concentrations of 63.46 mg/l (3.3 ng/10^7 ^platelets) for PDE2, 225 mg/l (11.7 ng/10^7 ^platelets) for PDE3 and 1359 mg/l (70.7 ng/10^7 ^platelets) for PDE5. Total molar concentrations are calculated based on a platelet volume of 5.2 fl as reported in [14].

Using the experimentally determined PDE concentrations and assuming that 80% of total cAMP turnover is provided by PDE3 activity [16] simulations from the basic model of the unstimulated platelet indicate that basal cyclic nucleotide levels cannot be maintained with these levels of active PDEs but are rapidly diminished (Additional file 1, Figure S2.1). In fact, mathematical calculations yielded a cAMP hydrolysis activity of 1846.1 μM/min for the PDE3 isoform (75% of total activity) as well as 642.9 μM/min for PDE2 (~ 25% of total activity). This would give total cAMP hydrolysis rate of 2.5 mM/min corresponding to a turnover rate of the entire platelet cAMP pool of 625 times per minute. These results suggest that not the entire amount of PDE is enzymatically active but rather the majority of the enzyme remains inactive in resting platelets. Parameter optimization constraining on a constant basal cAMP level of 4 μM yielded an enzymatically active PDE concentration of 0.05 mg/l for PDE2 (only 0.1% of total PDE2 concentration) and 2.3 mg/l for PDE3 (just 1% of total PDE2 concentration), respectively. This precisely reproduced cyclic nucleotide levels under resting conditions (Additional file 1, Figure S2.1). Consequently, this predicts an *in vivo *activity of 1.5 μM/min for PDE2 and of 6.5 μM/min for PDE3, yielding a total cAMP hydrolysis activity of 8 μM/min. This agrees with apparently low available concentrations suggested by electron microscopy (Additional file 1, Part III), though high total PDE concentrations were measured *in vitro*. The low active concentration for the antibody stain may be due to e.g. enzyme sequestration or inactivation in a complex (epitope masking) [20]. Thus, either substantial amounts of cAMP degrading PDEs are not catalytically active and/or the *in vivo *enzyme activity is significantly lower than under *in vitro *conditions. Furthermore, there is experimental evidence indicating the compartmentalization of PDE3 and PDE5 [20-22]. To incorporate all experimental findings, we thus extended the model to include equilibrium between an active or inactive PDE form (Figure 1, Figure 3).

### Model validation using experimental time series data from cAMP elevating compounds

After the development of the basal model of cyclic nucleotide signaling under resting conditions, we experimentally analyzed and modeled pathway effects of drugs causing elevated cyclic nucleotide levels in platelets thus inhibiting platelet aggregation (anti-platelet effect).

**Figure 4:**
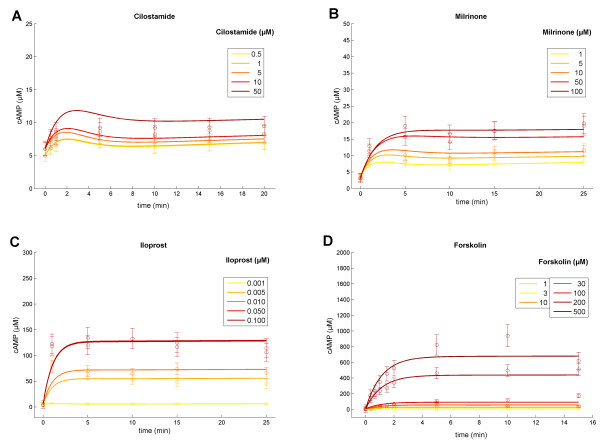
**Multi-experimental data fit**. Means of three independent cAMP measurements (circles) and estimated standard error of the mean, resulting of cAMP elevating platelet treatments as PDE3 inhibition **(A, B) **and activation of adenylyl cyclase **(C, D) **through administration of varying drug doses. Displayed curves reflect predicted model trajectories of the optimized pathway models: Models for PDE inhibition and AC activation were fit simultaneously to all displayed data (of number data points *N =*155; *χ*^2 ^= 129.08), by precisely estimating a set of only 23 parameters in total. Top: Inhibition of PDE3 with different concentrations of Cilostamide **(A) **and Milrinone **(B) **Bottom: Model trajectories and measurements after adenylyl cyclase stimulation with different concentrations of Iloprost **(C) **and Forskolin **(D)**. Colors: Varying concentrations of given drug stimuli (hardly any cAMP elevating effect of Cilostamide at low administered doses).

We here focus on the cAMP pathway, probing varying drug doses of PDE inhibitors and stimulators of AC: The inhibition of PDE3 by Milrinone (1, 5, 10, 50, 100 μM) or Cilostamide (0.5, 1, 5, 10, 50 μM) resulted in a delayed increase in cAMP concentration (Figure 4A, B, respectively), whereas Milrinone exerted a larger effect resulting in higher cAMP levels compared to those induced by treatment with comparable doses of Cilostamide. Stimulation of the prostacyclin receptor by Iloprost (1, 5, 10, 50, 100 nM, Figure 4C) or allosteric activation of ACs by Forskolin (1, 3, 10, 30, 100, 200, 500 μM) evoked an immediate and pronounced cAMP increase (Figure 4D). Dosage-dependent stimulation by the stable prostacyclin analog Iloprost led to elevated cAMP plateaus with saturation beyond the administration of 50 nM Iloprost (Figure 4C). Similarly, stimulation by diterpene Forskolin (500 μM) achieved high cAMP concentrations and a plateau (700 μM cAMP) after five minutes (Figure 4D). For each time point and drug dosage, three independent cAMP measurements were quantified.

### Model selection: Multi-experiment fitting and testing hypotheses on cAMP feedback loops

To incorporate all experimental findings, we iteratively expanded and refined the established basal model by fitting the resulting models reflecting PDE inhibition and AC stimulation simultaneously to all collected data sets:

We therefore modified the enzymatic catalysis (PDE3) by incorporating the kinetics for competitive inhibition (Figure 3B) as reported in [23] resulting in the observed Michaelis-Menten-like reaction rate

(1) $$\upsilon =\frac{V_{\max}\left(PDE3\right)\cdot c\left(PDE3\right)\cdot c\left(cAMP\right)}{\left(1.0+\frac{u_{i}}{k_{i}}\right)\cdot K_{m}\left(PDE3\right)+c\left(cAMP\right)}.$$

Here, *u_i _*represents the concentration of the inhibitor (Milrinone, Cilostamide), whereas *k_i _*denotes the respective inhibition constant. These drug-specific constants adjust the apparent K_m_-value of the competitively inhibited enzyme.

To model AC activation, we introduce additional rates

(2) $$\upsilon =x_{Forskolin},\;\upsilon =x_{Iloprost}$$

respectively, mimicking the increased cAMP formation by AC (Figure 3C). This rate has been estimated locally for each concentration of the AC activators Forskolin and Iloprost.

We conducted and evaluated fit sequences by slightly disturbing all model parameters before again adapting the model to time series data. We chose fitting ranges of unknown parameters (Additional File 1, Part II) according to mean values derived from literature if available (e.g. basal cAMP influx, inhibition constants) or selected a broad range in effect not limiting the parameter space during fitting (influx rates). Multi-experiment fitting sequences provided us with *χ*^2 ^values, indicating the quality of model fits (Table 1). This reveals that the adapted model, according to equations (1) and (2), could not explain all experimental data. Addressing this discrepancy, we considered two possibilities to further expand and refine the model: Feedback regulation of the cAMP level is known to be mediated through activation of PDE3 and inhibition of AC, which both have been experimentally observed [24-28]. The mechanism of PDE3 activation is also indicated by possible PDE3 phosphorylation at Ser312, a PKA phosphorylation site, as reported in PlateletWeb [12].

**Table 1:** Evaluation of integrated feedback loops within the dynamic model.

Hypotheses	*Χ* ^2^	*N*	*p*	*Χ*^2^/*N*	*AIC*
H0	530.19	155	21	3.42	857.04
H1	185.52	155	22	1.2	514.395
H2	133.00	155	22	0.9	461.825
H3	129.08	155	23	0.8328	459.952

Competing hypotheses: **H0 **- no integrated assumption of feedback loops; **H1 **- positive feedback of cAMP-dependent PKA to PDE3 (one additional parameter); **H2 **- negative feedback of elevated cAMP levels to AC (one additional parameter); **H3 **- both assumptions (**H1**, **H2**) are integrated within the mathematical model (two additional parameters). For each evaluated hypotheses the resulting *χ*^2 ^-value, number of data points (*N*), number of estimated model parameters (*p*) and the Akaike information criterion (*AIC*) are listed.

Therefore, we modified the underlying model equations (1) and (2) by introducing a cAMP-dependent increase of *V*_max_(*PDE*3)

(3) $$V_{\max}new=V_{\max}\left(PDE3\right)+kf_{1}\cdot c\left(cAMP\right).$$

Factor *kf*_1 _thereby weights the cAMP feedback resulting in an activation of PDE3.

Analogous, we added a cAMP-dependent term mimicking AC inhibition in rate equations (2) for *i *= Forskolin and Iloprost, respectively:

(4) $$\upsilon_{new}=x_{i}-kf_{2}\cdot c\left(cAMP\right).$$

Feedback constant *kf*_2 _adjusts the strength of this negative feedback loop.

We investigated by likelihood ratio tests for competing nested pairs of models [29] whether both model refinements are statistically necessary to expand the model structure. Table 2 shows the resulting p-values for testing hypotheses on both feedback loops. We tested either positive feedback of cAMP-dependent PKA on PDE3 activity (H1), negative feedback of PKA inhibiting AC activity (H2) or both feedback loops simultaneously (H3) against the null hypothesis that the parameter (*kf*_1_, *kf*_2_) with regard to each feedback loop equals zero (H0). Albeit, assuming one feedback solely (H1 or H2) enhanced the overall model fit (p < 0.05), it failed in explaining all time series simultaneously. For a given level of significance of 0.05, likelihood ratio tests of nested models evidenced a significantly better fit to the data by including both (i) the inhibition of AC, as well as (ii) the cAMP-dependent activation of PDE3 by PKA (H3 vs H2, p = 0.0477; H3 vs H1, p = 5.80·10^-14^). Hence, the inclusion of both feedback constants statistically led to the most reasonable model structure. Modeled accordingly, this resulted in model trajectories explaining all data simultaneously (Figure 4A-D). The trajectories do not differ significantly from the experimental data (*χ*^2^/*N *= 0.8328<1; *N *= 155 number of data points) by estimating a small set of 23 specific parameters (Additional file 1, Section 7). This modeling approach considers the full set of time series data resulting from PDE inhibition and AC stimulation experiments at varying drug dose ranges, allowing for hypotheses testing of inherent feedback loops.

**Table 2:** Hypothesis testing and model selection.

Test scenario	p-value
H1 versus H0	<10^-15^
H2 versus H0	<10^-15^
H3 versus H1	5.80·10^-14^
H3 versus H2	0.0477

Four competing hypotheses on feedback loop integration (see Table 2) are tested with regard to the given level of significance (0.05). For each scenario, the appropriate likelihood ratio test for nested model pairs was conducted (based on *χ*^2 ^-values of resulting model fit sequences). This reveals that the integration of both additional feedback constants is statistically relevant to explain all time series data on cAMP elevating drugs.

The validated *in silico *model reflects processes of platelet activation and models correctly the experimental measurements by considering low effective PDE concentrations, activation of PDE3 by PKA at increased cAMP levels as well as the negative feedback loop of cAMP-dependent PKA inhibiting AC activity.

### Parameter sensitivity

We analyzed several characteristics of the platelet cAMP signal, indicating the state of inhibition, to investigate the influence of model parameters on this inhibitory signal. Results exemplified for one PDE3 inhibitor (Cilostamide) and one AC stimulator (Iloprost) indicate that in case of PDE3 inhibition (low and high dose; Additional file 2, Figure S1.1A, B), the cAMP signal is mainly controlled by the basal formation of cAMP. The impact of drug-specific and PDE-specific constants augments with the increase of the inhibitory drug dose. A similar sensitivity profile for parameters can be observed for low-dose AC stimulation (Additional file 2, Figure S1.1C) whereas at high doses, particularly the feedback constant *kf*_2 _(AC inhibition) gains importance and outweighs the influence of basal cAMP formation (Additional file 2, Figure S1.1D). This corroborates the pivotal role of feedback regulation of the cyclic nucleotide level in platelets.

To highlight the effect of the feedback constants *kf*_1 _(equation (3)) and *kf*_2 _(equation (4)) on the model predictions for cAMP signal, constants were perturbed (multiplication with factors ranging from 0.1 to 10). Constant *kf*_1 _(feedback activation of PDE3) resulted in cAMP signal spans ranging from 3 to 5 μM (Additional file 2, Figure S1.2A, B), however, this range expanded up to 300 μM (500 μM Forskolin). The cAMP signal is highly sensitive to AC feedback inhibition (constant *kf*_2_) even at low concentration (1 nM) of Iloprost (Additional file 2, Figure S1.2C). This is also reflected in the model selection procedure (Table 2) again indicating a subtle feedback regulation of cAMP levels.

### Combination effects of different drugs

**Figure 5:**
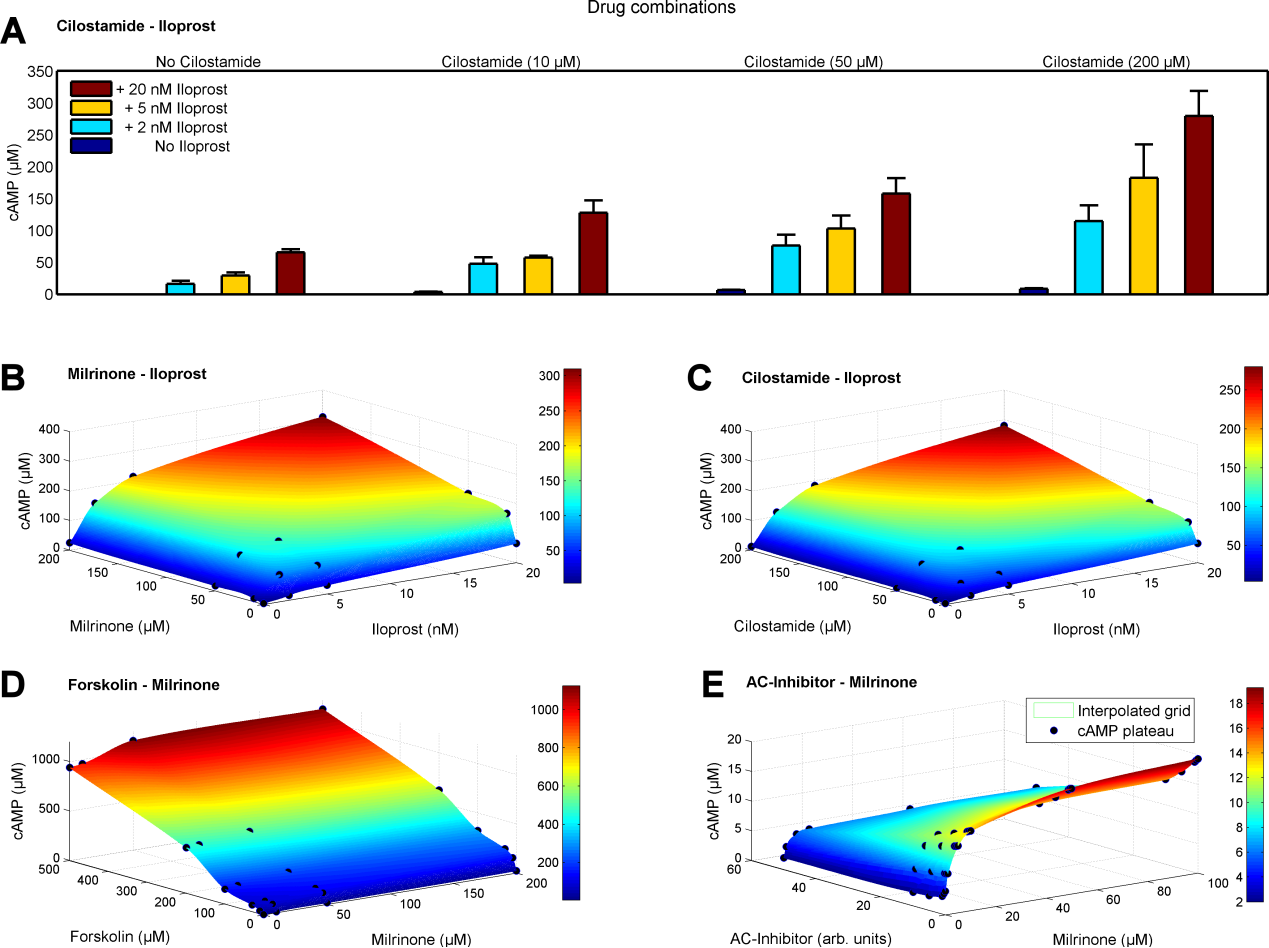
**Predicted time course and experimentally measured cAMP levels under varying drug combination conditions**. Besides modeling results **(B, D, E)**, panel **A **and **C **show experimentally measured cAMP levels (marked with black dots). Single and combinatory effect of Cilostamide (drug doses range: 10, 50, 200 μM) and Iloprost (drug doses range: 2, 5, 20 nM) on the cAMP level **(A)**: The resulting cAMP concentrations, depicted as the mean of triplicate measurements (± SEM), successively rise according to the corresponding elevated drug doses and show a clear synergistic effect. 3D-plots show interpolated cAMP levels (z-axis; cAMP surface) of estimated or experimentally measured plateaus of cAMP reached at various synergistically activating or inhibitory drug combinations (x- and y-axes): Combination **(B) **of Iloprost and Milrinone (AC activation, PDE inhibition); **(C) **of Iloprost and Cilostamide (AC activation, PDE inhibition); **(D) **of Forskolin and Milrinone (AC activation, PDE inhibition); **(E) **of Milrinone together with an unspecified inhibitor of adenylyl cyclase (simultaneous PDE and AC inhibition). Dots mark means of experimentally measured cAMP level plateaus **(C) **and accordingly predicted cAMP values **(B, D, E) **reached after the respective chosen concentration and combination of drugs was administered. For further details on drug combination interactions see Additional file 1, Table S8.1.

Having established a validated dynamic model on the basis of time series data of four different anti-platelet drugs at varying concentrations, we can use this model to predict and to study drug combination effects in detail. Here we investigate the effects of simultaneous applications of PDE3 inhibitors (Milrinone and Cilostamide) and AC activators (Iloprost and Forskolin). In particular, we analyze the combined effect of Iloprost and Cilostamide by measuring experimentally the response to each drug individually (Cilostamide: 10, 50, 100 μM; Iloprost: 2, 5, 20 nM) as well as the effect of combinations of all drug doses on the cAMP level (Figure 5A). The experimentally measured cAMP plateaus for each drug combination are depicted in Figure 5C (black dots). This clearly reveals an over-additive effect of the two drugs when applied in combinations. Based on this, we predict cAMP levels of other drug combinations (Figure 5B-E), illustrating the capabilities of the full model: Simultaneous stimulation of AC and inhibition of PDE3 is modeled by combining Iloprost (2, 5 and 20 nM) and Milrinone (10, 50 and 200 μM; Figure 5B) or Cilostamide, respectively (Figure 5C). The surface of cAMP level results from interpolating the reached cAMP plateaus (black dots) at the respective drug combination; the origin marks the basal cAMP level, each axis indicates the effect of the single drugs.

As synergistic effects of PDE inhibitors in combination with Iloprost have been described in human platelets [30-32] we embedded an additional cAMP-dependent constant *k *to capture this over-additive effect (Additional file 1, Section 8). In adaptation to quantitative data of experiments combining Cilostamide and Iloprost, we find the calculated cAMP level surface in close accordance to measured cAMP concentrations (Figure 5C), resulting in *k *= 1.443 ± 0.004. This way, the predicted cAMP levels describe perfectly the subsequent experimental measurements in contrast to assuming an additive effect of drug combinations (p < 0.05). This indicates a continuous synergistic effect of Cilostamide and Iloprost interaction potentiating the respective single drug stimulation (Figure 5A).

Furthermore we analyzed the combined effects of Forskolin and Milrinone (Figure 5D) by predicting cAMP levels of combining Forskolin drug doses (10, 30, 100, 200, 500 μM) with Milrinone doses of 10, 50 and 200 μM. The surface of cAMP level interpolates calculated cAMP level plateaus reached at the distinct combinations of drug. Similarly, we predict cAMP levels for the simultaneous inhibition of AC and PDE3, respectively. Figure 5E shows the successive decrease of elevated cAMP level due to Milrinone (1-100 μM) by combining this PDE3 inhibition with several doses of an AC inhibitor (e.g. 2'5'Dideoxyadenosine; non-synergistic, *k *= 1). In addition, we give a summary and classification of typical platelet drugs (Additional file 1, Table S8.1) allowing computer based modeling of drug (combination) effects.

### Pathway integration: Phosphorylation of individual VASP phosphorylation sites

**Figure 6:**
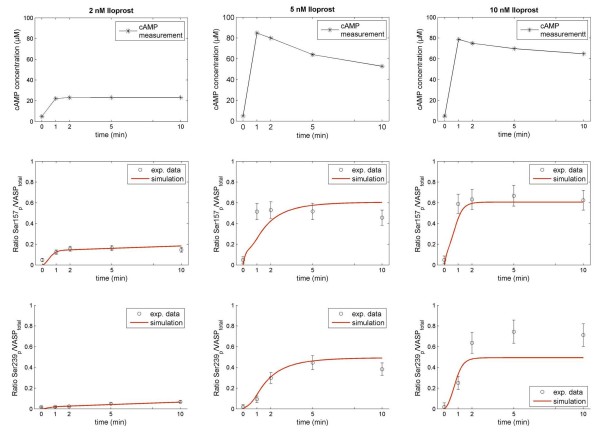
**Time-resolved VASP phosphorylation upon administration of cAMP elevating drug doses**. Ratios of phosphorylated VASP sites (Ser157, Ser239) to the total amount of VASP in platelets are measured after administration of doses of AC stimulator Iloprost (2 nM, 5 nM, 10 nM) at distinct time points (black circles ± calculated SD). The experimentally measured cAMP time course (top panel of each row), marked with black stars serves as model input. Phosphosite Ser157 is phosphorylated prior to Ser239 by PKA as indicated by data and model (red trajectories).

An important downstream effector of cyclic nucleotide signaling is VASP. This phosphoprotein has two major phosphorylation sites (Ser157 and Ser239) [33] which both negatively regulate platelet aggregation. Since this protein is a major target for both, PKA (cAMP pathway) and PKG (cGMP pathway), it can integrate the input from these two pathways. Thus, it is a paradigm for complex combination effects regarding phosphorylation events. Here we consider and model the differential effect of PKA on both of these sides. Site Ser157 is a 15-fold better substrate for phosphorylation by PKA than the phosphosite Ser239 being also a substrate for the cGMP-dependent protein kinase PKG [33,34]. Furthermore, this differential phosphorylation of PKA has to be considered for the read-out of diagnostic VASP assays [35]. The phosphosite-specific modeling results in a good fit to experimental measurements (Figure 6) and allows again predictions for different drug strengths and combinations (Additional file 1, Section 5, 6).

### Cross-talk between cyclic nucleotide signaling pathways

**Figure 7:**
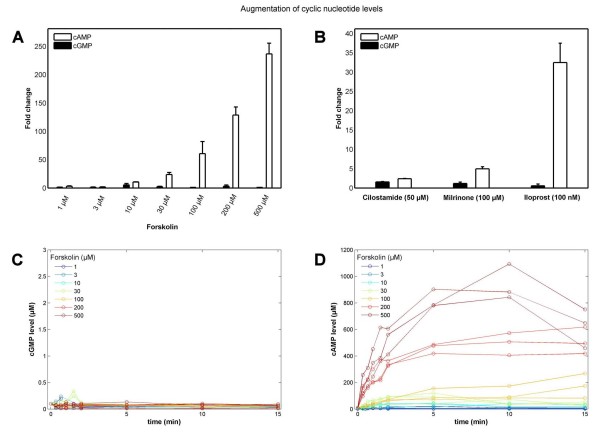
**No cross-talk from cAMP to cGMP pathway**. **(A) **Bar plot of fold changes (mean of three measurements ± SEM) of different cyclic nucleotide levels for cAMP (white) and cGMP (black) with respect to basal cyclic nucleotide levels upon Forskolin stimulation (1-500 μM). Strong stimulation by Forskolin leads to high cAMP levels **(A, D) **by activating AC yet does not change low cGMP levels **(C)**. Similarly, high doses of Cilostamide (50 μM), Milrinone (100 μM) and Iloprost (100 nM) elevate the cAMP level solely, leaving cGMP levels unaffected **(B)**.

Interestingly, experimental data and model predictions evidence that the cAMP response in platelets is highly specific: No cross-talk to the cGMP pathway for cAMP stimulating compounds can be observed regarding change in cGMP levels (Figure 7). Even at cAMP concentrations up to the millimolar range e.g. due to exceeding AC stimulation by Forskolin (500 μM) the cGMP levels remain unaffected (Figure 7A, C-D). Furthermore, PDE3 inhibition by Cilostamide (50 μM) and Milrinone (100 μM) as well as AC stimulation (Iloprost, 100 nM) solely elevate the cAMP level (Figure 7B). Regarding a cAMP-focused pathway model based on stimulation of AC (e.g. by Forskolin, Iloprost) or inhibition of cAMP-degrading PDEs (e.g. by Milrinone, Cilostamide), the cGMP-specific components of the model (PDE5, GC and their related parameters) can thus be neglected.

### Probing the signaling network sensitivity

To investigate the network sensitivity, we considered different perturbations and network cross-linking: We analyzed the model performance by probing drug stimuli (inhibition of PDE2 and PDE3) of different time-scales (transient vs. long-tem; concurrent vs. successive).

The network sensitively responds to transient stimuli even at low stimuli doses (Additional file 2, Figure S2.1). The same holds for transient prostaglandin receptor activation [35]. Physiologically, this receptor is transiently activated by prostacyclin (half-life of less than 5 minutes [36]), produced by the endothelium. Thus, in case of an injury of the vessel wall parts of the endothelium remain without the production of prostacyclin so that only an insufficient signal is invoked and the platelet lacks inhibition. Similarly, patho-physiological conditions like prostacyclin receptor mutations [37,38] contribute to a differential platelet inhibition decreasing the protection against unwanted platelet activation and aggregation. Modified platelet activation is crucial for thrombus self-organization and the formation of thrombi. We showed this gradual transient platelet inhibition via different prostacyclin doses as model response (cAMP) as well as experimentally, by considering cAMP-dependent, time-resolved phosphorylation events on VASP (Ser157) (Additional file 2, Figure S2.2).

Furthermore, we showed that an elevation of platelet cAMP level has no effect on the cGMP pathway, however the converse cross-talk has been described [1,39,40] and mainly mediated though cross-linking of the platelet phosphodiesterases. We therefore speculated on the effect of cGMP stimuli by incorporating identified PDE interconnections into the model structure (Additional file 2, Figure S2.3).

## Discussion

### Mathematical modeling of platelet signaling

ODE-based dynamic models have emerged as fruitful tool for the modeling of biological systems and signaling pathways [8,11,41,42]. In particular, they allow data-based kinetic modeling and the detailed investigation of pharmacological effects on cellular signaling cascades. In general, this works best for moderate-sized networks where sufficient detailed data are available to estimate model parameters. In this sense, the cyclic nucleotide signaling pathway in human platelets is especially well-suited for such an approach as there is enough information on the kinetics available and the number of key components is rather moderate.

Here, we present a mathematical model, based on existing biological knowledge and an extensive set of experimental measurements. Using the powerful approach of multi-experiment fitting, we optimized the model parameters in the context of several *in vitro *data sets. Since this provides a higher number of data points for the estimation of parameters, it permits not only the estimation of predictive model parameters but also statistically verifies the validity of a given model. Moreover, this allows for discrimination between competing model hypotheses [43,44]. With this approach we could use the entire set of N = 155 experimental data points (triplicates) to obtain reliable estimates of the basic model parameters by globally fitting them to the dataset.

### Pathway simulations and integration

Models on thrombosis or endothelial function have been published before [6,7], but so far none investigated the cAMP mediated signaling in the platelet in detail. In this study, we established a time-resolved *in silico *model of fine-tuned cAMP signaling in platelets. The data and model can serve as a basis to gain a deeper insight into the basic effects of platelet cyclic nucleotide signaling. Predictions based on simulations of the basal *in silico *model indicate that only a very small fraction of the PDEs is enzymatically active under basal conditions which might be a result of PDE compartmentalization as has been reported for adipocytes (PDE3), HEK293 cells and cardiac myocytes (PDE4) and platelets (PDE5) [20-22,45].

Elevated levels of cyclic nucleotides potently contribute to platelet inhibition targeting cyclic nucleotide-dependent protein kinases that in turn phosphorylate specific substrates. Via cAMP degrading phosphodiesterases, cGMP can regulate the level of cAMP in cardiac cells including platelets [1,46]. Within this context, we show system state transitions from the non-activated cAMP pathway in platelets to an activated state as well as activatory and inhibitory cross-linking directed from the cGMP and the cAMP pathway (Additional file 1, Figure S1.2). Key feedback regulations involve the resting state of the platelet and the activation of both the cAMP pathway and cGMP pathway. Interestingly, the cAMP pathway is highly specific: Even at considerably high cAMP levels no cross-talk to the cGMP pathway could be observed. This is validated here including experimental measurements.

Furthermore, we investigated how the two branches of cyclic nucleotide signaling in human platelets might be integrated. Therefore, we focused on VASP, a prominent PKA and PKG substrate and highly connected hub protein. Signal integration by VASP protein relies on the activation of the downstream cytoskeletal regulating signaling cascade [35,47]. Taking into account the two major known PKA/PKG phosphorylation sites, Ser157 and Ser239, we accurately predict and experimentally validate resulting differential activation in quantitative terms of specific PKA mediated phosphorylations on VASP.

### Effects of drug combinations

Platelets play a critical role in thrombosis and hemostasis. Therefore, it is important that no accidental activation of platelets occurs and the intricate balance between activation and inhibition is maintained. This has implications for pharmacological fine-tuning of the system. Various pharmacological substances affecting cAMP signaling in platelets are in clinical use. The most prominent mechanisms of anti-platelet drugs are the inhibition of P2Y12 receptors (Clopridogrel, Prasugrel, Ticlopidine), of COX1 and thromboxane (Acetylsalicyclic acid) and of phosphodiesterases (Dipyridamole, Cilostazol, Milrinone) as well as antagonistic effects on integrin αIIbβ3 (Tirofiban, Eptifibatide, Abciximab) [48,49]. Other drugs such as antagonists of platelet prostaglandin receptor EP3 for prostaglandin E2 targeting platelet G-protein-coupled receptors (GPCRs) or adenylyl cyclases as potential drug targets are under development [4,50]. Furthermore, anti-platelet therapy benefits of the potential over-additive effect of platelet drugs applied in combination [48,51] and of the investigation of drug interactions [52] potentially leading to a decrease in drug dosage and undesirable side effects like bleeding. Hence, a more detailed understanding of the interactions and effects of these substances on platelet activation and inhibition is not only of scientific interest but also of clinical importance. Having calibrated the model with experimental data of different drugs, the final model allows predictions not only about the effect of single drugs but also about combinations thereof. A major challenge of drug interaction modeling is to account for synergistic, additive or antagonistic drug interactions [53,54]. This can be achieved by individual parameters for each specific combination of drug doses resulting in different types of interactions becoming apparent on the cAMP level. Concerning the modeled drug interactions, we succeeded in modeling these different modes of drug interaction with just one unique cAMP dependent parameter. The assumed simplification led to reliable and robust predictions compatible with the observed experimental data. However, we are aware that more complex effects and different modes of action have to be introduced if a broader range of drugs is considered and larger data sets are available.

### Feedback regulation of cAMP level

Moreover, the system analysis based on experiments of platelet stimulation with anti-platelet drugs points to an important role of AC and PDE feedback primarily from PDE3. Focusing on major pathway components it becomes clear that these two feedback loops are to be involved to achieve optimal signal strengths. Induced by elevated cAMP concentration, activated PKA inhibits AC but as well activates PDE3 [25-27]. Short-term feedback regulation of cyclic nucleotide concentrations resulting from activation of cAMP-dependent PKA have been indicated in various cell types (rat hepatocytes, adipocytes, myoblasts, smooth muscle cells, osteoclasts, U937 cells) including platelets [24,26,55-59] showing the ubiquitous nature of this mechanism. Here, we focused on the activation of PDE3 as well as the inhibition of AC activity, both in PKA-dependent manner.

The activation of PDE3A and PDE4A by PKA has been observed in smooth muscle cells [26]. Moreover, PDE3A is phosphorylated by PKA at Ser312 in a response to Forskolin [60] resulting in PDE3A activation in human platelets [24]. Our model results suggest a more prominent role for AC inhibition by PKA phosphorylation. This PKA-dependent inhibitory effect has been described for adenylyl cyclase subtypes 5 (AC5) and 6 (AC6) in smooth muscle cells [26]. As platelet proteomics data indicate the expression AC5 and AC6 besides AC subtype 3 and 7 in human platelets, direct phosphorylation of AC5 or AC6 by PKA might contribute to the feedback inhibition of cAMP synthesis [25,28].

Our data suggest that both feedback effects are crucial for regulating cAMP levels in human platelets where the inhibitory effect on AC outweighs the positive feedback effect on PDE3 (Table 2).

## Conclusions

We established a comprehensive dynamic model using multi-experiment fitting procedures, which enables us to evaluate and test questions of pharmacological interest. Our *in silico *model integrates central cAMP regulating mechanisms for a comprehensive description of cAMP signaling in human platelets. It has been tested and successfully applied to compare synergistic and non-synergistic as well as activatory and inhibitory drug combination effects on this fine-tuned platelet signaling pathway through which signals are sensitively propagated. The inhibitory cAMP pathway is well balanced: Our modeling approach reveals low active PDE concentrations compared to those experimentally measured in the platelet as well as two feedback loops allowing highly reliable signal transmission.

High sensitivity for low effector concentrations, threshold behavior and stable platelet inhibition are resulting. Perturbations such as prostacyclin receptor mutations and varying time scales of drug stimulation (transient vs. constant, successive vs. concordant) are modeled as well to probe the network sensitivity. In contrast to cGMP, which is known to interact with the cAMP pathway [40], converse cross-talk of cAMP to the cGMP pathway was neither predicted nor observed. However, direct integration over signals from cAMP and cGMP pathways is achieved via the integrator protein VASP and its phosphorylation sites, thereby also monitoring the effects of anti-platelet drugs.

Future work will extend the model to cover more complex scenarios such as stimulation of Gi-coupled receptors (e.g. P2Y12) which are known to decrease cAMP levels in human platelets thereby leading to platelet activation. Further model development will concern the integration of cGMP pathway effects on platelet cAMP levels upon stimulation of GCs.

In perspective, the *in silico *modeling approach of platelet cAMP as well as cGMP regulation will support future drug development as well as strategies for anti-aggregatory treatment and provides a unique tool for experimental design of pharmacological studies of platelets.

## Methods

### Platelet preparation

Washed human platelets [61] were prepared as described [62]. Briefly, whole human blood was drawn after informed consent by venipuncture from volunteers who had not received any platelet or cyclic nucleotide affecting drugs in the past two weeks. Blood was collected in weakly acidic citrate/dextrose buffer (4:1 v/v). Whole blood was centrifuged at 300 xg for 10 min at 20°C. The platelet rich plasma obtained was centrifuged (380 xg, 20 min at 20°C). The resulting platelet pellet was resuspended in HEPES buffer (145 mM NaCl, 5 mM KCl, 1 mM MgCl_2_, 10 mM HEPES, 10 mM glucose, pH7.4) to a final cell density of 10^8^/ml. Experiments were carried out by incubation of the platelet suspension at 37°C with the respective compounds and for the time. All experiments were reproduced with at least three different preparations.

### Chemicals

Forskolin (Sigma, Munich, Germany), Iloprost (Schering, Berlin, Germany), Milrinone (Sigma, Munich, Germany) and Cilostamide (Tocris Cookson, Avonmouth, UK) were used as indicated in the results. PDE2A (C-terminal fragment aa1-207 deletion, C-terminal His6-tag), PDE3A (aa675-1164 fragment, N-terminal GST-tag) and PDE5A (full length, C-terminal His6) were a kind gift by J. A. Beavo, University of Seattle, Washington, USA.

### Measurements of cAMP/cGMP levels

The stimulation was stopped by addition of twice the sample volume of ice cold ethanol (100%). The precipitate was removed by centrifugation, washed again with 100 μl ethanol; the supernatants were combined and dried in the vacuum. Dried platelet samples were dissolved, acetylated and analyzed with commercial cAMP or cGMP enzyme-immuno assays (ENZO Life Sciences, Loerrach, Germany) according to manufacturer's instructions.

### Measurements of PDE concentrations

PDE2 and PDE5 were determined from calibration curves with lysates from recombinant expression systems [34]. In brief, *E. coli *BL21-T1 strain was transformed with plasmids containing inserts coding for PDE2A, PDE3A or PDE5A and lysed after IPTG induction. The lysates were then purified by affinity chromatography. The GST tagged PDE3A fragment was bound to a GST-Sepharose column (Glutathione Sepharose 4B, Amersham Biosciences) and eluted according to the manufacturers' protocol. The His6-tagged PDE2A and PDE5A were purified on a Ni-NTA column (Ni-NTA Superflow Kit, Quiagen) and eluted according to the manufacturers' protocol. The protein content of the eluents was determined by the BCA assay (bicinchoninic acid, BCA protein assay, Thermo Scientific) and dilution series prepared. Platelet lysates and the dilution series of recombinant protein were then submitted to acrylamide gel-electrophoresis, transferred on a nitrocellulose membrane and detected with an appropriate antibody and chemoluminescence reaction (ECL plus, Amersham). The intensity of the protein bands was quantified after scanning with ImageJ software (v1.43, NIH) and the platelet expression determined from the calibration curves obtained from the dilution series. PDE2 and PDE5 were determined from calibration curves with lysates in over-expression systems [34]. PDE3 amount was taken from [14], kinetic and binding data from [34,63].

### VASP phosphorylation/immunofluorescence

VASP phosphorylation was determined for both phosphorylation sites according to [35] with the phosphosite specific antibodies 16C2 (pS239) or 5C6 (pS157) and the total VASP antibody IE273. Stimulated washed platelet suspensions were stopped by addition of lysis buffer (20 mM Tris-HCl (pH 7.4), 150 mM NaCl, 1 mM EDTA, 1 mM EGTA, 1% Triton X-100, 0.5% NP-40, 10 mM ß-glycerolphosphate, 10 mM NaF) and frozen. The frozen samples were thawed and diluted with PBS buffer (137 mM NaCl, 2.7 mM KCl, 10 mM Na_2_HPO_4_, 2 mM KH_2_PO_4_, pH = 7.4) by 1:10. As control for background and non-specific binding 5% bovine serum albumin (BSA) solved in lysis buffer was used. Each sample was measured in triplicate for each antibody. VASP was immobilized from the sample by binding on a zyxin coated microtiter plate and incubation for 1 h at room temperature under shaking. The microtiter plates were washed three times with 300 μl/well PBS-T (1% Triton X-100 supplemented PBS) and the primary antibodies were added (5 μg/ml for the phosphospecific antibodies and 1 μg/ml for IE273) in 100 μl portions and again incubated for 1 h and washed 3 times with PBS-T. Detection of antibody binding was achieved with horse radish peroxidase (HRP) coupled goat anti-mouse IgG as secondary antibody and 100 μl of the HRP substrate ABTS (2,2'-azino-bis(3-ethylbenzothiazoline-6-sulfonic acid) diammonium salt) dissolved in 4 ml ABTS buffer (Roche) and diluted 1:10 with aqua dest. The absorbance of the samples was measured in the microtiter plates with a Wallac Victor 1420TM (Perkin-Elmer) plate reader at 405 nm each for 1 second. From the absorbance of each sample, the absorbance of the background control sample for the respective antibody was subtracted and the data expressed as relative phosphorylation calculated by dividing the absorbance signal obtained with the respective phosphospecific antibody by the signal obtained with the IE273 antibody.

### *In silico *modeling

A set of ordinary differential equations (ODEs) represent platelet cAMP and cGMP signaling pathways (Additional file 1, Part II). Rate constants taken from literature all arise from studies on human platelets [12-20,24,27], inhibition constants are from different systems to delimit their fitting ranges. The basal model incorporated Mass Action and Michaelis Menten kinetics (Additional file 1, Part II, Section 2, Appendix). It was next optimized by fitting it simultaneously and experiment-specific to data of PDE inhibition experiments (Additional file 1, Part II, Section 3), AC stimulation experiments (Additional file 1, Part II, Section 4) as well as to phosphorylation data of VASP phosphosites (Additional file 1, Part II, Section 5, 6). Corresponding differential equations were implemented and further analyzed using the MATLAB software (The Mathworks, Inc. Natick, MA).

### Parameter optimization

Estimating model parameters which optimize the *χ*^2 ^-merit function and set the model statistically compliant with the data is a crucial problem [43]. For integrating the dynamic model and optimal parameter estimation we used the MATLAB toolbox PottersWheel [43] including multi-experiment fitting. To fit the model $y=y\left(t,\vec{p}\right)$ to data, we optimize the *χ*^2 ^*-*merit function $\chi^{2}\left(\vec{p}\right)=\sum {\left(\left(y_{i}-y\left(t_{i},\vec{p}\right)\right)/\sigma_{i}\right)}^{2}$,with *y_i _*being data point *i *with standard deviation *σ_i _*and $y\left(t_{i},\vec{p}\right)$describes the model value at time point *i *for a set of parameter values $\vec{p}$. In case of normally distributed measurement errors, this corresponds to a maximum likelihood estimation. For optimizing this function, we used the trust region algorithm [44], a powerful deterministic least-square optimizer. This did yield optimal parameters $\vec{p}$ for modeling e.g. the platelet effector experiments, minimizing the distance between model trajectories and time series data.

### Model selection as hypothesis testing

For selecting an adequate model structure, being the most crucial part of the modeling process, we conduct the following forward strategy: We start with the most parsimonious reasonable model and refine it iteratively and directed by biochemical knowledge until subsequent refinement does not significantly improve the model fitting process. Therefore, we conducted a commonly used method for model comparison, the likelihood ratio test (LRT) comparing pairs of nested models characterized by a different number of parameters [29]. Assuming a more complex model M*_C _*with *p_C _*parameters competes with the simpler model M*_S _*having *p_S _*inherent parameters. Then, the likelihood ratio (twice the difference of the log likelihoods L) is distributed as 2 (L(M*_C_*)-L(M*_S_*))~*χ*^2 ^with *p_C_*-*p_S _*degrees of freedom, which penalizes overparameterization. Given a chosen level of significance, this enables to determine whether the data supports the simple model (null hypothesis) or if the more complex model offers a sufficient improvement.

## Appendix: Basic model - Mathematical formalization

Here, we discuss the basic model and its features in detail. This model, consisting of a system of ordinary differential equations is constructed to simulate the basal concentrations of cyclic nucleotides assuming resting platelet conditions.

### Reaction scheme

Unstimulated platelets hold specific basal levels of the cyclic nucleotides cAMP and cGMP [14]. There is a constant afflux of cyclic nucleotides arising from the corresponding adenylyl and guanylyl cyclase, respectively [17-19]. Active phosphodiesterases PDE2, PDE3 and the cGMP-specific PDE5 counterbalance their activity by enzymatically degrading cyclic nucleotides [2,16]. The kinetics of these enzyme reactions is well understood as well as the cyclase activities. Thus, this prior biochemical knowledge frames the basis for setting up the basal signaling network as well as the underlying reactions as indicated in Figure 3A.

### Dynamic variables and constants

For this, we formalize dynamic variables and constants listed in Table 3. All values are known from the literature except specific concentration values of the phosphodiesterases. However, this enables us to mathematically estimate and compare these PDE concentrations with those experimentally determined.

**Table 3:** Dynamic variables and constants of basal cyclic nucleotide signaling.

Model parameter	Values	Foundations/assumptions	Reference
*Dynamic variables*			

*x*_1_: c(cAMP)	4 μM	Basal levels	[14]
*x*_2_:c(cGMP)	0.4 μM	Basal levels	[14]

*x*_3_:c(PDE2) active	0.05 mg/l	Model-based simulation	This study
		Experimentally: 63.46 mg/l	
*x*_4_:c(PDE3) active	2.3 mg/l	Model-based simulation	This study
		Experimentally: 225 mg/l	
*x*_5_:c(PDE5) active	1 mg/l	Model-based simulation	This study
		Experimentally: 1359 mg/l	
*x*_6_:c(PDE2) inactive	(63.46 -*x*_3_) mg/l		
*x*_7_:c(PDE3) inactive	(225 - *x*_4_) mg/l		
*x*_8_:c(PDE5) inactive	(1359 - *x*_5_) mg/l		

*x*_9_:c(AMP)	μM; simulated		
*x*_10_:c(GMP)	μM; simulated		

*Constants*			

*k*_1_: V_max _PDE2	120 μmol/min/mg	cAMP turnover	[2,16]
*k*_2_:K_m _PDE2	50 μM	cAMP turnover	[2,16]
*k*_3_:V_max _PDE3	3 μmol/min/mg	cAMP turnover	[2,16]
*k*_4_:K_m _PDE3	0.2 μM	cAMP turnover	[2,16]

*k*_5_:V_max _PDE2	120 μmol/min/mg	cGMP turnover	[2,16]
*k*_6_:K_m _PDE2	35 μM	cGMP turnover	[2,16]
*k*_7_:V_max _PDE3	0.3 μmol/min/mg	cGMP turnover	[2,16]
*k*_8_:K_m _PDE3	0.02 μM	cGMP turnover	[2,16]
*k*_9_:V_max _PDE5	5 μmol/min/mg	cGMP turnover	[2,16]
*k*_10_:K_m _PDE5	5 μM	cGMP turnover	[2,16]

*k*_11_:kcAMP	8 μmol/min	Basal influx of cAMP	[17-19]
*k*_12_:kcGMP	1 μmol/min	Basal influx of cGMP	[64,65]

*k*_13_:Deactivation PDE2			This study
*k*_14_:Activation PDE2	*k*_13_-*k*_18_: = 0		This study
*k*_15_:Deactivation PDE3	for simulation of		This study
*k*_16_:Activation PDE3	basal levels		This study
*k*_17_:Deactivation PDE5	(resting conditions)		This study
*k*_18_:Activation PDE5			This study

*k*_19_:hPDE2	2	Hill coefficient	[15,16]

Description of the model parameters and constants of the formalized the system of ODEs modeling the basal cyclic nucleotide signaling (see Appendix).

### Kinetics and reaction rates

For model simulations and predicting PDE concentrations, we assume Mass Action kinetics for the basal generation of cyclic nucleotides (reaction *r*1,*r*2) and the PDE-dependent degradation (reactions *r*3*-r*7) are described by Michaelis Menten kinetics. In particular, cAMP shows positively cooperative kinetic effects, resulting in a Hill coefficient of 2 regarding PDE2 catalytic activity [15,16]. By means of the error model 0.1·*y+*0.05·max(*y*) calculating the standard deviation depending on measurements *y *of the observables cAMP and cGMP, respectively, we simulate the basal cyclic nucleotide levels by estimating PDE concentrations (PottersWheel toolbox). Model-based simulations reveal comparatively low PDE concentrations with respect to the experimentally determined PDE levels that fail in maintaining a basal level but abolish them immediately (Additional file 1, Figure S2.1). Biochemically motivated, we thus include a possible switch from the active to an inactive PDE state (reactions *r*8-*r*13) assuming Mass Action. This results in reaction rates *υ*_1_-*υ*_13 _for all modeled reactions *r*1-*r*13 listed in Table 4.

**Table 4:** Modeled reactions and rates of basal cyclic nucleotide signaling.

Reaction	Rate
Basal AC influx of cAMP (*r*1):	*υ*_1 _= *k*_11_;
Basal GC influx of cGMP (*r*2):	*Υ*_2 _= *k*_12_;
cAMP turnover via PDE2 (*r*3):	*$\upsilon_{3}=k_{1}\cdot {x_{1}}^{k_{19}}\cdot x_{3}/\left(k_{2}+{x_{1}}^{k_{19}}\right);$*
cAMP turnover via PDE3 (*r*4):	*υ*_4 _*= k*_3_·*x*_4_*·x*_1_/(*k*_4_+*x*_1_);
cGMP turnover via PDE2 (*r*5):	*υ*_5 _*= k*_5_·*x*_3_*·x*_2_/(*k*_6_+*x*_2_);
cGMP turnover via PDE3 (*r*6):	*υ*_6 _*= k*_7_·*x*_4_*·x*_2_/(*k*_8_+*x*_2_);
cGMP turnover via PDE5 (*r*7):	*υ*_7 _*= k*_9_·*x*_5_*·x*_2_/(*k*_10_+*x*_2_);
(De)activation of PDE (*r*8- *r*13):	*υ*_8 _*= k*_14_·*x*_6_;
	*υ*_9 _*= k*_13_·*x*_3_;
	*υ*_10 _*= k*_16_·*x*_7_;
	*υ*_11 _*= k*_15_·*x*_4_;
	*υ*_12 _*= k*_18_·*x*_8_;
	*υ*_13 _*= k*_17_·*x*_5_;

Itemized modeled reactions *r *and rates *υ *describing the basal cyclic nucleotide signaling (see Appendix).

This leads to the final system of differential equations of the dynamic variables *x*_1_-*x*_10 _as described in Table 3. A Systems Biology Markup Language file of this basal model is provided (Additional file 3) as well as its extension to the overall model (Additional file 4).

### Differential equations

$$dx_{1}/dt=+\upsilon_{1}-\upsilon_{3}-\upsilon_{4};$$

$$dx_{2}/dt=+\upsilon_{2}-\upsilon_{5}-\upsilon_{6}-\upsilon_{7};$$

$$dx_{3}/dt=+\upsilon_{8}-\upsilon_{9};$$

$$dx_{4}/dt=+\upsilon_{10}-\upsilon_{11};$$

$$dx_{5}/dt=+\upsilon_{12}+\upsilon_{13};$$

$$dx_{6}/dt=-\upsilon_{8}+\upsilon_{9};$$

$$dx_{7}/dt=-\upsilon_{10}+\upsilon_{11};$$

$$dx_{8}/dt=-\upsilon_{12}+\upsilon_{13};$$

$$dx_{9}/dt=+\upsilon_{3}+\upsilon_{4};$$

$$dx_{10}/dt=+\upsilon_{5}+\upsilon_{6}+\upsilon_{7}^{};$$

## List of abbreviations

cAMP: cyclic adenosine monophosphate; AMP: adenosine monophosphate; cGMP: cyclic guanosine monophosphate; GMP: guanosine monophosphate; AC: adenylyl cyclase; GC: guanylyl cyclase; PDE: phosphodiesterase; PKA: cAMP-dependent protein kinase; PKG: cGMP-dependent protein kinase; VASP: vasodilator stimulated phosphoprotein; GPCR: G-protein-coupled receptor; ODE: ordinary differential equation; SD: standard deviation; LRT: likelihood ratio test; AIC: Akaike information criterion; SEM: standard error of the mean.

## Authors' contributions

GW, MD designed and performed the mathematical modeling. MD, GW, EB, JG and TD analyzed data and improved iteratively the model. EB, RM, KH, JG did the experiments. TD drafted the manuscript; MD, GW, EB, JG and TD were involved in writing. JG led and supervised the experimental part of the project. TD led the project and supervised the computational work. All authors read and approved the final manuscript.

## Supplementary Material

Additional file 1 **Supplementary Information**. The supplementary information is divided into three parts. Part I (**S1**) deals with the model topology, pathway cross-linking and gives information about the main components of the modeled cAMP- and cGMP signaling pathways (Table S1.1). The second part (**S2**) provides detailed information about the mathematical modeling including variables and constants, reaction schemes and rates as well as systems of differential equations. Sections 3-6 deal with the modeling of the following scenarios: PDE inhibition via Cilostamide and Milrinone (Section 3), adenylyl cyclase activation via Forskolin and Iloprost (Section 4) and finally downstream phosphorylation of VASP (Section 5, 6). The fitted parameters are listed in Section 7 (Table S7.1), information about modeling of drug combinations and specific parameters of drugs being crucial for the examined platelet signaling cascades are given in Section 8 (Table S8.1). Section 9 introduces the established SBML-models of cyclic nucleotide signaling (Additional file 3, 4). An electron microscopy micrograph of PDE is depicted in Part III (**S3**). Click here for file

Additional file 2 **Additional Results: Network sensitivity**. Additional results: Sensitivity analysis and probing of the network sensitivity (permanent and transient model perturbations and pathway cross-linking). Click here for file

Additional file 3 **This SBML model file encodes the basal model**. A Systems Biology Markup Language file representing the basal model of cyclic nucleotide signaling. This model is implemented with CellDesigner (Version 4.0.1) for simulating the basal cyclic nucleotide levels under resting conditions. All kinetic parameters and concentration values are specified within this file. Click here for file

Additional file 4 **SBML model file encoding the overall model**. Comprehensive Systems Biology Markup Language file implemented with CellDesigner (Version 4.0.1) for investigating and simulating cyclic nucleotide levels under the designated conditions. In addition to Additional file 3, this model file contains signaling nodes regarding the downstream events (VASP phosphorylations) as well as anti-platelet drugs. Click here for file
