# Supplementary material for: Time-resolved in silico modeling of fine-tuned cAMP signaling in platelets: feedback loops, titrated phosphorylations and pharmacological modulation
Source: BMC Syst Biol. 2011 Oct 28;5:178. doi: 10.1186/1752-0509-5-178 (PMC3247139; doi:10.1186/1752-0509-5-178)
Supplement: Additional file 1 — Supplementary Information. The supplementary information is divided into three parts. Part I (S1) deals with the model topology, pathway cross-linking and gives information about the main components of the modeled cAMP- and cGMP signaling pathways (Table S1.1). The second part (S2) provides detailed information about the mathematical modeling including variables and constants, reaction schemes and rates as well as systems of differential equations. Sections 3-6 deal with the modeling of the following scenarios: PDE inhibition via Cilostamide and Milrinone (Section 3), adenylyl cyclase activation via Forskolin and Iloprost (Section 4) and finally downstream phosphorylation of VASP (Section 5, 6). The fitted parameters are listed in Section 7 (Table S7.1), information about modeling of drug combinations and specific parameters of drugs being crucial for the examined platelet signaling cascades are given in Section 8 (Table S8.1). Section 9 introduces the established SBML-models of cyclic nucleotide signaling (Additional file 3, 4). An electron microscopy micrograph of PDE is depicted in Part III (S3). [file 1752-0509-5-178-S1.PDF]

Additional file 1 for:

Time-resolved *in silico* modeling of fine-tuned cAMP signaling in platelets: feedback loops, titrated phosphorylations and pharmacological modulation

Gaby Wangorsch[1], Elke Butt[2], Regina Mark[2], Katharina Hubertus[2],
Jörg Geiger[2], Thomas Dandekar[*,1,3], Marcus Dittrich[1]

1 Department of Bioinformatics, Biocenter, University of Würzburg, Am Hubland, 97074 Würzburg, Germany.
2 Institute for Clinical Biochemistry & Pathobiochemistry, Grombühlstraße 12, 97080 Würzburg, Germany
3 EMBL, Postfach 102209, 69012 Heidelberg, Germany

* Corresponding author: Thomas Dandekar, phone: 0931-888-4551. FAX -4552;
E-mail: dandekar@biozentrum.uni-wuerzburg.de

Short introduction to supplementary information:

The supplementary information is divided into three parts.
Part I (**S1**) deals with the model topology (**S1**, Fig S1.1) and gives information about the main components of the modeled cAMP- and cGMP signaling pathways (**S1**, Table S1.1) and Fig S1.2 illustrates possible pathway cross-linking.
The second part (**S2**) provides detailed information about the mathematical modeling including variables and constants, reaction schemes and rates as well as systems of differential equations. Sections 3-6 deal with the modeling of the following scenarios: Phosphodiesterase (PDE) inhibition via Cilostamide and Milrinone (Section 3), adenylyl cyclase activation via Forskolin and Iloprost (Section 4) and finally downstream phosphorylation of VASP (Section 5,6). The fitted parameters are listed in Section 7 (Table S7.1), information about modeling of drug combinations and specific paramters of drugs being crucial for the examined platelet signaling cascades are given in Section 8 (**S2**, Table S8.1). Section 9 introduces the established SBML-models of cyclic nucleotide signaling.
Part III (**S3**) shows an electron microscopy micrograph of PDE.

Contents

I S1 Cyclic nucleotide signaling cascades - general information 3

1 Model topology, components and pathway cross-linking 3

II S2 Details on model establishment 6

2 Basal level of cyclic nucleotides in platelets 6

3 PDE inhibition via Cilostamide/Milrinone 7
3.1 Reaction scheme 7
3.2 Variables and constants (PDE inhibition model) 8
3.3 Reaction rate formalisms 9
3.4 System of differential equations 9

4 Adenylyl cyclase (AC) activation via Forskolin/Iloprost 10
4.1 Reaction scheme 10
4.2 Variables and constants (AC activation model) 11
4.3 Reaction rate formalisms 11
4.4 System of differential equations 11

5 VASP phosphorylation 1 - PKA and PKG 12
5.1 Reaction scheme 12
5.2 Variables and constants 13
5.3 Rules 14
5.4 Reaction rate formalisms 14
5.5 System of differential equations 14
5.6 Experimental data compared to model trajectories (VASP phosphorylation model 1) 15

6 VASP phosphorylation 2 - two distinct catalytic PKA subunits 16
6.1 Reaction scheme 16
6.2 Variables and constants 17
6.3 Rules 18
6.4 Reaction rate formalisms 18
6.5 System of differential equations 18
6.6 Experimental data compared to model trajectories (VASP phosphorylation model 2) 19

7 Data-driven parameter fitting 20

8 Parameters for drug combinations 21

9 SBML Files 22

III S3 Electron microscopy 24

Part I
S1 Cyclic nucleotide signaling cascades - general information

1 Model topology, components and pathway cross-linking

Model topology

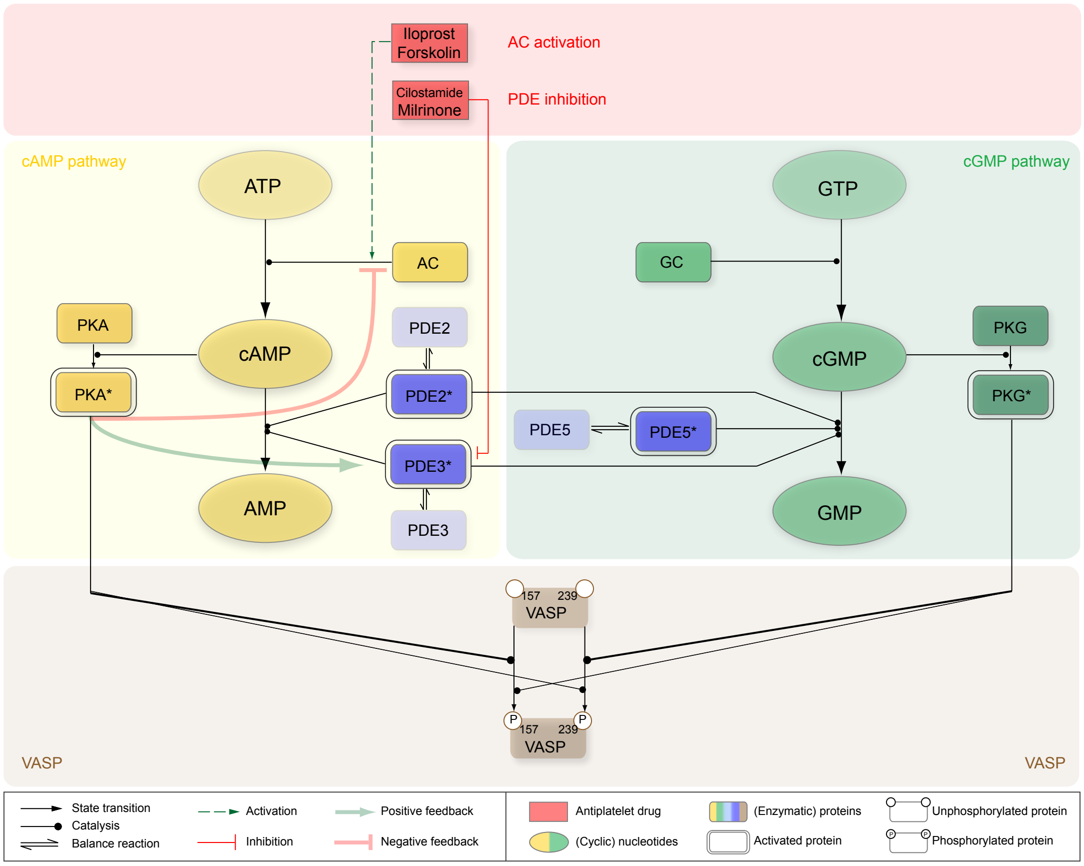

Fig S1.1 Topology of modeled signaling cascades. The cAMP signaling cascade is depicted in yellow, the cGMP pathway in green. Influencing drugs, relevant for the modeling scenarios, are illustrated in the red part (PDE inhibitors, AC stimulators) together with their functions (activation: green arrow; inhibition: red arrow). Downstream events (VASP phosphorylations) are shown in brown; PKG mainly phosphorylates VASP at Ser239 (bold arrow). The same is true for PKA and the Ser157 site.

Model components

Table S 1.1 Model components and specific parameter values.

Component, parameter	Parameter Values	Reference	Remark
**Cyclases and cyclic nucleotides**			
**Adenylate cyclase (AC)**		HPRD, Plateletweb	Not explicitly modeled,
ADCY7, ADCY3, ADCY6			No SAGE tags for any AC isoform
**Basal AC activity**	15.9 pmol/mg/min=7.16 μM/min	[1]	Assumption: Constant influx of cAMP
	12.4 pmol/mg/min=5.6 μM/min	[2]	in unstimulated platelets
	0.0376 amol/min/platelet=7.23 μM/min	[3]	
**cAMP**	4.4 $\pm$ 1.0 μM/min	[4]	Basal level in platelets
**Guanylyl cyclase (sGC)**		HPRD, Plateletweb	Not explicitly modeled,
GUCY1A3, GUCY1B3			No SAGE tags for any AC isoform
**Basal sGC activity**	0.6 - 1 μM/min	[5]	Assumption: Constant influx of cGMP
			in unstimulated platelets
**cGMP**	0.4 $\pm$ 0.1 μM/min	[4]	Basal level in platelets
**Phosphodiesterases (PDEs)**			
**PDE2A**, cGMP stimulated		HPRD	cGMP stimulated, increase in activity
(allosterically)			at physiological concentrations (1-10 μM),
			no increase in V_{max}.
			Inhibited by high cGMP concentrations
			(beyond 20 μM) by competition[1]
PDE2-specific parameters:		[6]	
		[7]	
PDE2 concentration	63.46 mg/l	This study	Assumption: 0.05 mg/l
Hill coefficient	2		Homodimer with hill coefficient of 2
K_m-value (cAMP turnover)	50 μM		
V_{max}-value (cAMP turnover)	120µmol/min/mg		
K_m-value (cGMP turnover)	35 μM		
V_{max}-value (cGMP turnover)	120 µmol/min/mg		
**PDE3A**, cGMP-inhibitied		HPRD	80% of cAMP PDE activity is provided
			by PDE3 ([6]),
			cGMP inhibited (competitive inhibition; $IC50 = K_m$)[1]
			Phosphorylation by PKA increases PDE3 activity
			→ responsible for basal cAMP level
PDE3-specific parameters:		[6]	
		[7]	
PDE3 concentration	225 mg/l	This study	Assumption: 2.3 mg/l
K_m-value (cAMP turnover)	0.2 μM		
V_{max}-value (cAMP turnover)	3 µmol/min/mg		
K_m-value (cGMP turnover)	0.02 μM		Not modeled[1]
V_{max}-value (cGMP turnover)	0.3 µmol/min/mg		Not modeled[1]
**PDE5A**, cGMP specific		HPRD	Only minor role in model so far
PDE5-specific parameters:		[6]	
PDE5 concentration	1359 mg/l	This study	Assumption: 1 mg/l in basal model, otherwise[1]
K_m-value (cGMP turnover)	5 μM		In basal model, otherwise[1]
V_{max}-value (cGMP turnover)	5 µmol/min/mg		In basal model, otherwise[1]
**PDE5B, PDE9A, PDE7A**		[8]	Evidenced expression albeit only at a very low level
			of one tag, → not modeled
**VASP**			
**VASP**		HPRD	
VASP-specific parameters:			
Phosphorylation sites:		[9]	Platelet: Ser157 is phosphorylated more
Ser157, Ser239, Thr278		[10]	rapidely by PKA, Ser239 more rapidly
			by PKG, Thr278 much weaker substrate
VASP concentration	25.2 $\pm$ 7.6 μM	[4]	Intracellular concentration in platelets
**Protein kinases (PKA/PKG)**			
**PKA** concentration	3.1 $\pm$ 0.6 μM	[4]	Intracellular (platelet) concentration
**PKG** concentration	7.3 $\pm$ 0.8 μM	[4]	Intracellular (platelet) concentration

[1] not modeled so far - no GC stimulation data available

Pathway cross-linking and system states

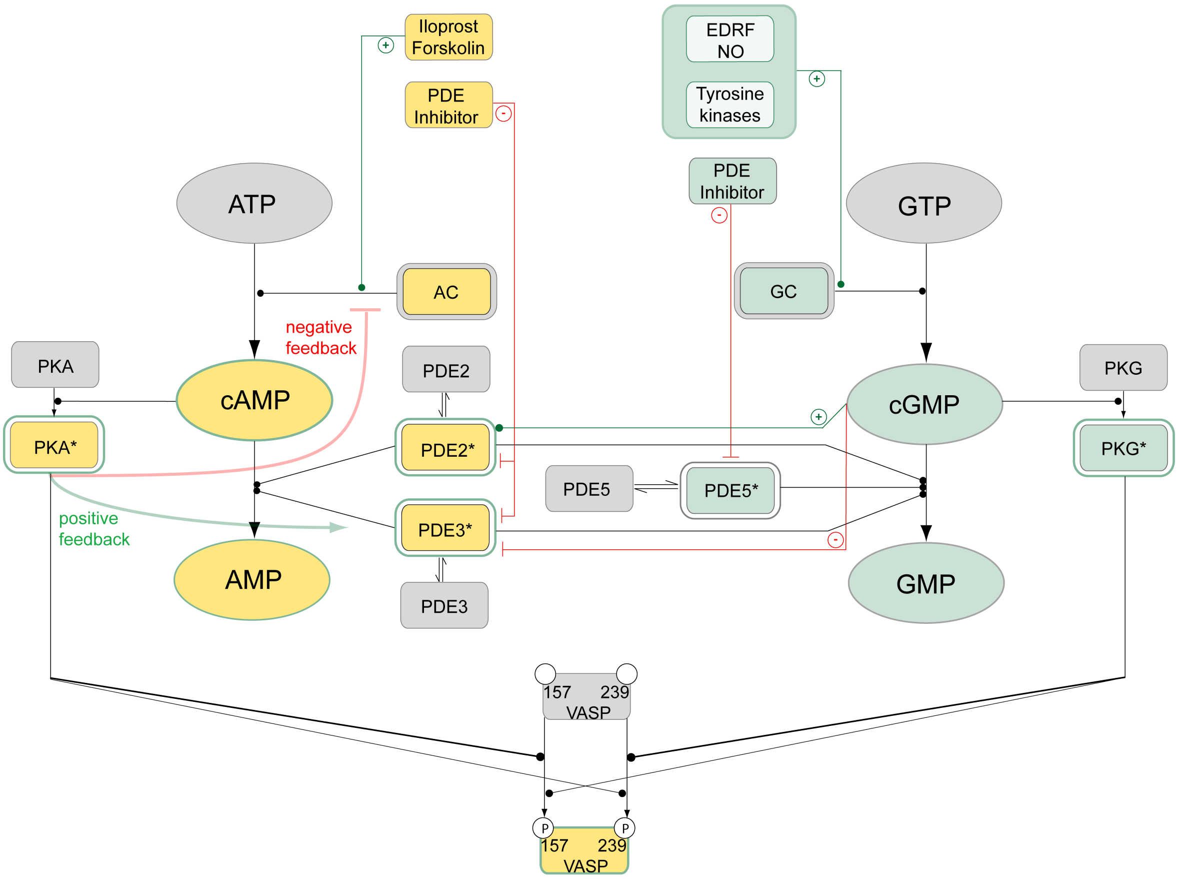

Fig S1.2 Comparison of different pathway states. Different system nodes involved in different cyclic nucleotide pathway stages: Resting state (gray nodes), activated cAMP path (yellow) and cGMP path (green) activated by endothelium-derived relaxing factor (EDRF) like nitric oxide (NO) or by tyrosine kinases and inhibitors of phosphodiesterase type 5. Components ubsequently activated via cGMP cross-talk are framed in green. Established feed forward loops (+) are depicted in green, negative feedback loops (-) in red with inhibition signs.

Part II
S2 Details on model establishment

2 Basal level of cyclic nucleotides in platelets

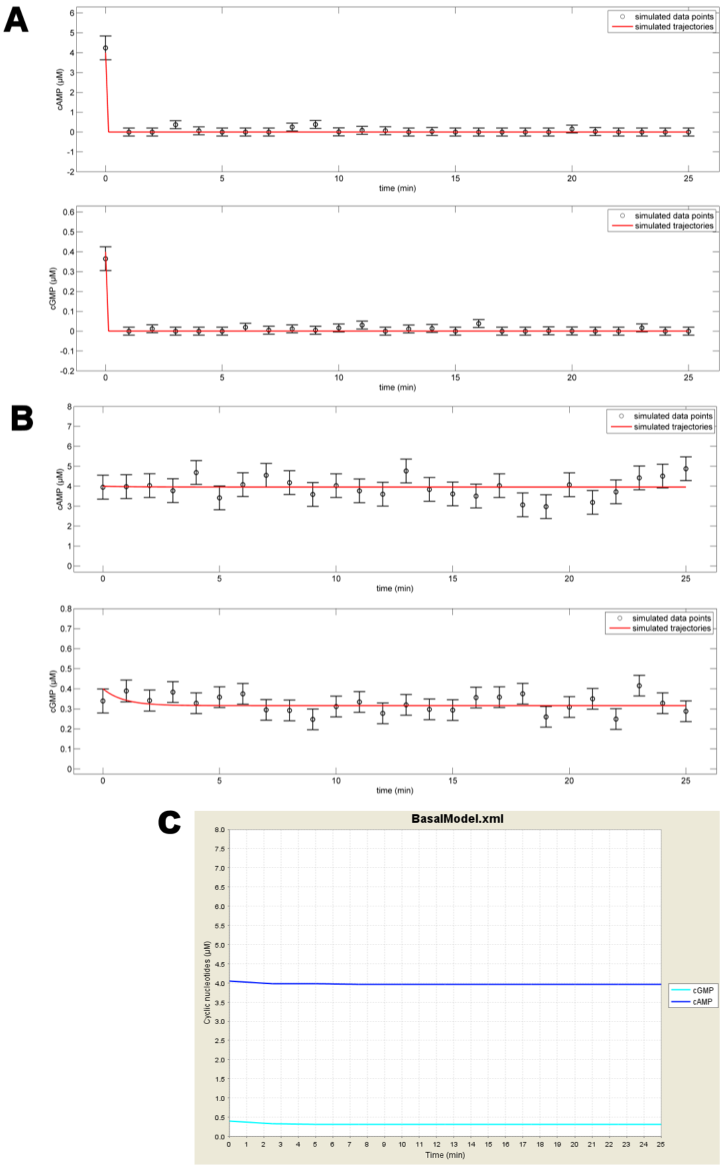

Fig S2.1 Simulations of basal levels of cyclic nucleotides in human platelets. Simulated time courses (25 min) for basal cyclic nucleotide levels (cAMP, cGMP) assuming (**A**) experimentally quantified total concentrations of PDE isoforms and (**B**) calculated PDE levels comprising prior knowledge of kinetic constants and PDE turnover rates Table S1.1. Black circles mark the simulated cAMP levels (y) at distinct time points with calculated SD (Gaussian distribution error: $0.10 * y + 0.05 * max(y)$). Red curves display the simulated model trajectories. If measured PDE concentrations were enzymatically active, this would fail to maintain the basal cAMP (4 μM) and cGMP (0.4 μM) levels in platelets but diminish them fast. In contrast, the basal concentrations are precisely reproduced by incorporating calculated levels of active PDE concentrations. Model simulation based on the SBML file (Additional file 3) assuming low active PDE concentrations (**C**).

3 PDE inhibition via Cilostamide/Milrinone

Building on the basal model (Appendix) and the pathway topology (part I) we consider here the modeling of PDE inhibition.
Variables and constants of modeled reactions (see Fig S3.1) are listed in Table S3.1. Details on reaction rates and the resulting system of differential equations are given in section 3.3 and 3.4.

3.1 Reaction scheme

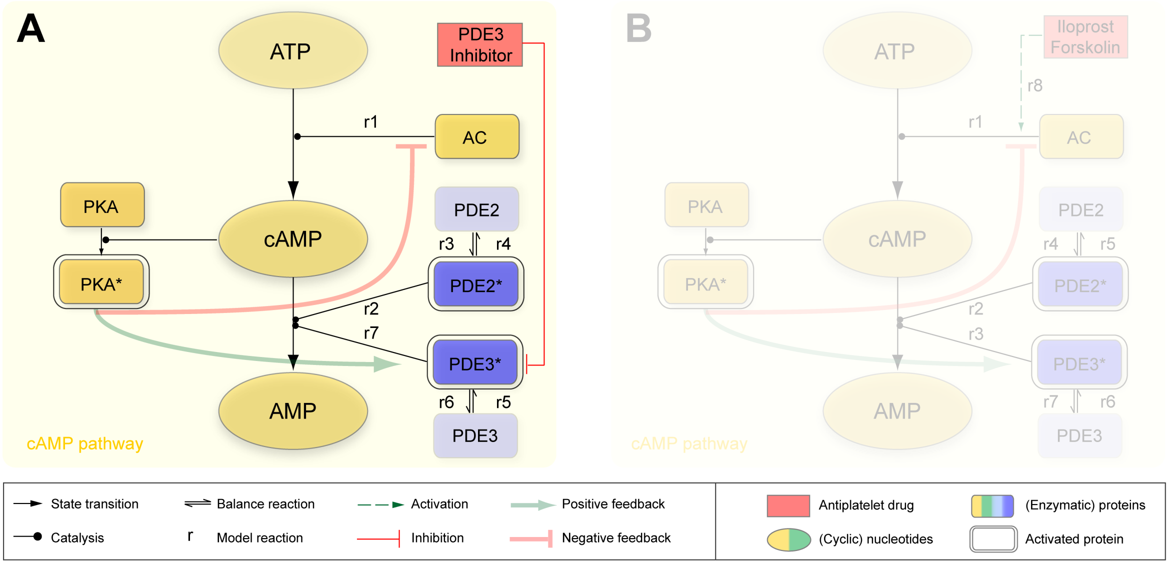

Fig S3.1 Reaction scheme of PDE3 inhibition - modeled reactions.

3.2 Variables and constants (PDE inhibition model)

Table S3.1 Set of variables and constants for mathematical model of PDE inhibition.

**Dynamic variables**	**Values** and **fitting range**	**Remarks**
x_1: c(PDE2) active	$[0.005, 0.2]$ mg/l	
x_2: c(PDE3) active	$[1.7, 3.5]$ mg/l	
x_3: c(PDE2) inactive	$[(63.46 - \text{c(PDE2)}), 63.46]$ mg/l	
x_4: c(PDE3) inactive	$[(225 - \text{c(PDE3)}), 225]$ mg/l	
x_5: c(cAMP)	µmol, simulated<br>Startvalue: 4 μM	
x_6: c(AMP)	μM	
**Input**		
u_1: c(Cilostamide)<br>u_2: c(Milrinone)	0.5, 1, 5, 10, 50 μM<br>1, 5, 10, 50, 100 μM	PDE3 inhibitor
**Constants**		
k_1: V_{max} PDE2	120 µmol/min/mg; fix	
k_2: K_m PDE2	50 μM; fix	cAMP turnover
k_3: V_{max} PDE3	3 µmol/min/mg; fix	
k_4: Feedback regulation	$[0, 0.2]$ µmol^{-1}	Activation of PDE3 via cAMP
k_5: K_m PDE3	0.2 μM; fix	cAMP turnover
k_6: kcAMP	$[5, 9]$ µmol/min	Basal influx of cAMP (AC)
k_7: hPDE2	2 ; fix	Hill coefficient (PDE2)
k_8: Deactivation of PDE2<br>k_9: Activation of PDE2<br>k_{10}: Deactivation of PDE3<br>k_{11}: Activation of PDE3	$[0, 1]$ min^{-1}	
k_{12_1}: k_i Cilostamide<br>k_{12_2}: k_i Milrinone	[0.00001, 1] μM	Inhibition constant (PDE3)

Parameter $x_1, x_2, k_4, k_6, k_8 - k_{12_2}$ fit to cAMP concentration measurements at several time points using parameter values given in Table S1.1.

3.3 Reaction rate formalisms

$$
\begin{aligned}
&\text{Basal AC influx of cAMP (r1)}: & v_1 &= k_6;\\
&\text{cAMP turnover via PDE2 (r2)}: & v_2 &= k_1 \cdot x_5^{k_7} \cdot x_1/(k_2 + x_5^{k_7});\\
&\text{(De)activation of PDE (r3-r6)}: & &\begin{cases} v_3 = k_9 \cdot x_3;\\ v_4 = k_8 \cdot x_1;\\ v_5 = k_{11} \cdot x_4;\\ v_6 = k_{10} \cdot x_2;\end{cases}\\
&\text{cAMP turnover via PDE3 (r7)}: & v_7 &= (k_3 + k_4 \cdot x_5) \cdot x_5 \cdot x_2/((1.0 + (u_i/k_{12_i})) \cdot k_5 + x_5);\\
& & &\text{with } u_i : \text{c(PDE3 inhibitor)}, \ k_{12_i} : \text{Inhibition constant}, i = 1, 2.
\end{aligned} \tag{3.1}
$$

3.4 System of differential equations

Variables are defined in Table S3.1.

$$
\begin{aligned}
\frac{dx_1}{dt} &= +v_3 - v_4;\\
\frac{dx_2}{dt} &= +v_5 - v_6;\\
\frac{dx_3}{dt} &= -v_3 + v_4;\\
\frac{dx_4}{dt} &= -v_5 + v_6;\\
\frac{dx_5}{dt} &= +v_1 - v_2 - v_7;\\
\frac{dx_6}{dt} &= +v_2 + v_7;
\end{aligned} \tag{3.2}
$$

4 Adenylyl cyclase (AC) activation via Forskolin/Iloprost

We next consider the modeling of activation of AC. Reoccurring variable names are equally to those in section 3. Similarly, variables and constants of modeled reactions (see Fig S4.1) are listed in Table S4.1. Reaction rates and the resulting system of differential equations are given in section 4.3 and 4.4.

4.1 Reaction scheme

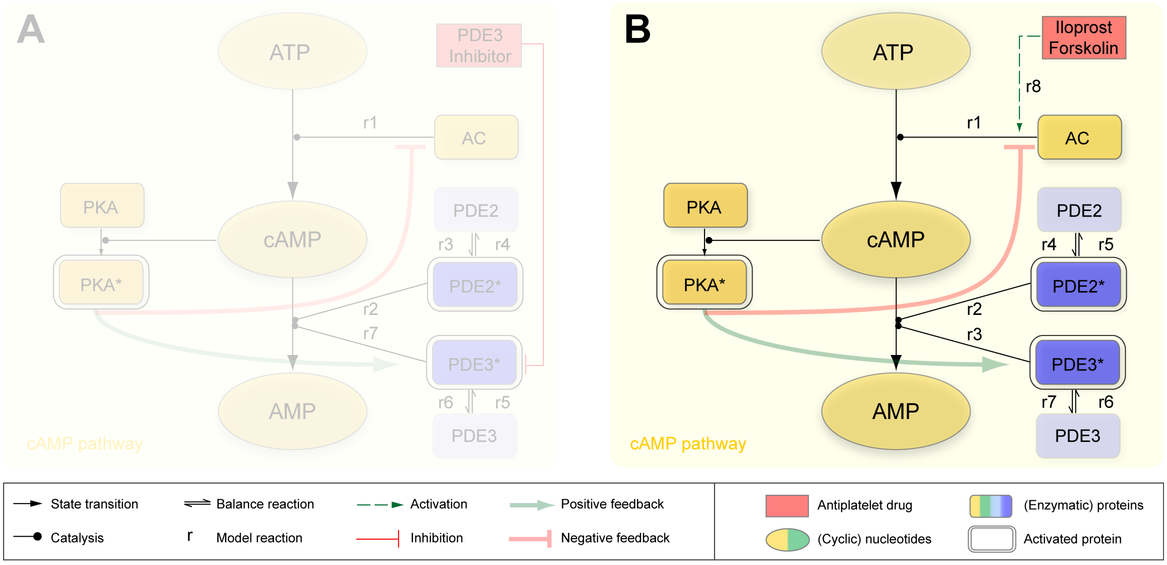

Fig S4.1 Reaction scheme of AC activation - modeled reactions.

4.2 Variables and constants (AC activation model)

Table S4.1 Set of variables and constants for mathematical model of adenylyl cyclase activation.

Dynamic variables	Values and fitting range	Remarks
x_1: c(PDE2) active	$[0.005, 0.2]$ mg/l	
x_2: c(PDE3) active	$[1.7, 3.5]$ mg/l	
x_3: c(PDE2) inactive	$[(63.46 - \text{c(PDE2)}), 63.46]$ mg/l	
x_4: c(PDE3) inactive	$[(225 - \text{c(PDE3)}), 225]$ mg/l	
x_{5_1}: $\text{AC}_{\textbf{Forskolin}}$	$[0, 5000]$ μmol/min	Response of Foskolin application (500, 200, 100, 30, 10, 3, 1 μM)
x_{5_2}: $\text{AC}_{\textbf{Iloprost}}$		Response of Iloprost application (100, 50, 10, 5, 1 nM)
x_6: c(cAMP)	μM, simulated<br>Startvalue: 4 μM	
x_7: c(AMP)	μM	
**Constants**		
k_1: V_{max} PDE2	120 μmol/min/mg; fix	
k_2: K_m PDE2	50 μM; fix	cAMP turnover
k_3: V_{max} PDE3	3 μmol/min/mg; fix	
k_4: Feedback regulation	$[0, 0.2]$ μmol^{-1}	Activation of PDE3 via cAMP
k_5: K_m PDE3	0.2 μM; fix	cAMP turnover
k_6: kcAMP	$[5, 9]$ μmol/min	Basal influx of cAMP (AC)
k_7: hPDE2	2 ; fix	Hill coefficient (PDE2)
k_8: Deactivation of PDE2		
k_9: Activation of PDE2	$[0, 1]$ min^{-1}	
k_{10}: Deactivation of PDE3		
k_{11}: Activation of PDE3		
k_{12_1}: Activation constant	1 min^{-1}; fix	Via Forskolin
k_{12_2}: of AC		Via Iloprost
k_{13}: Inhibition of AC	$[0, 10000]$ min^{-1}	Via cAMP (PKA)

Parameter $x_1, x_2, k_4, k_6, k_8 - k_{11}, k_{13}$ fit to cAMP concentration measurements at several time points using parameter values given in Table S1.1.

4.3 Reaction rate formalisms

$$
\begin{aligned}
&\text{Basal AC influx of cAMP (r1)}: && v_1 = k_6; \\
&\text{cAMP turnover via PDE2 (r2)}: && v_2 = k_1 \cdot x_6^{k_7} \cdot x_1/(k_2 + x_6^{k_7}); \\
&\text{cAMP turnover via PDE3 (r3)}: && v_3 = (k_3 + k_4 \cdot x_6) \cdot x_6 \cdot x_2/(k_5 + x_6); \\
&\text{(De)activation of PDE (r4-r7)}: && \begin{cases} v_4 = k_9 \cdot x_3; \\ v_5 = k_8 \cdot x_1; \\ v_6 = k_{11} \cdot x_4; \\ v_7 = k_{10} \cdot x_2; \end{cases} \\
&\text{cAMP influx via activated AC (r8)}: && v_8 = k_{12_i} \cdot x_{5_i} - k_{13} \cdot x_6; \\
& && \text{with } x_{5_i}: \text{AC}_{\textbf{Forskolin}}, \text{AC}_{\textbf{Iloprost}}, k_{12_i}: \text{Activation constant of AC}, i = 1, 2.
\end{aligned}
\tag{4.1}
$$

4.4 System of differential equations

Variables are defined in Table S4.1.

$$
\begin{aligned}
\frac{dx_1}{dt} &= +v_4 - v_5; \\
\frac{dx_2}{dt} &= +v_6 - v_7; \\
\frac{dx_3}{dt} &= -v_4 + v_5; \\
\frac{dx_4}{dt} &= -v_6 + v_7; \\
\frac{dx_5}{dt} &= 0; \\
\frac{dx_6}{dt} &= +v_1 - v_2 - v_3 + v_8; \\
\frac{dx_7}{dt} &= +v_2 + v_3;
\end{aligned}
\tag{4.2}
$$

5 VASP phosphorylation 1 - PKA and PKG

Next, we consider the modeling of VASP phosphorylation as downstream event (see Fig S5.1). Variables and constants of modeled reactions are listed in Table S5.1. Reaction rates, the resulting system of differential equations as well as time series data together with fitted model trajectories are given in sections 5.3-5.6.

5.1 Reaction scheme

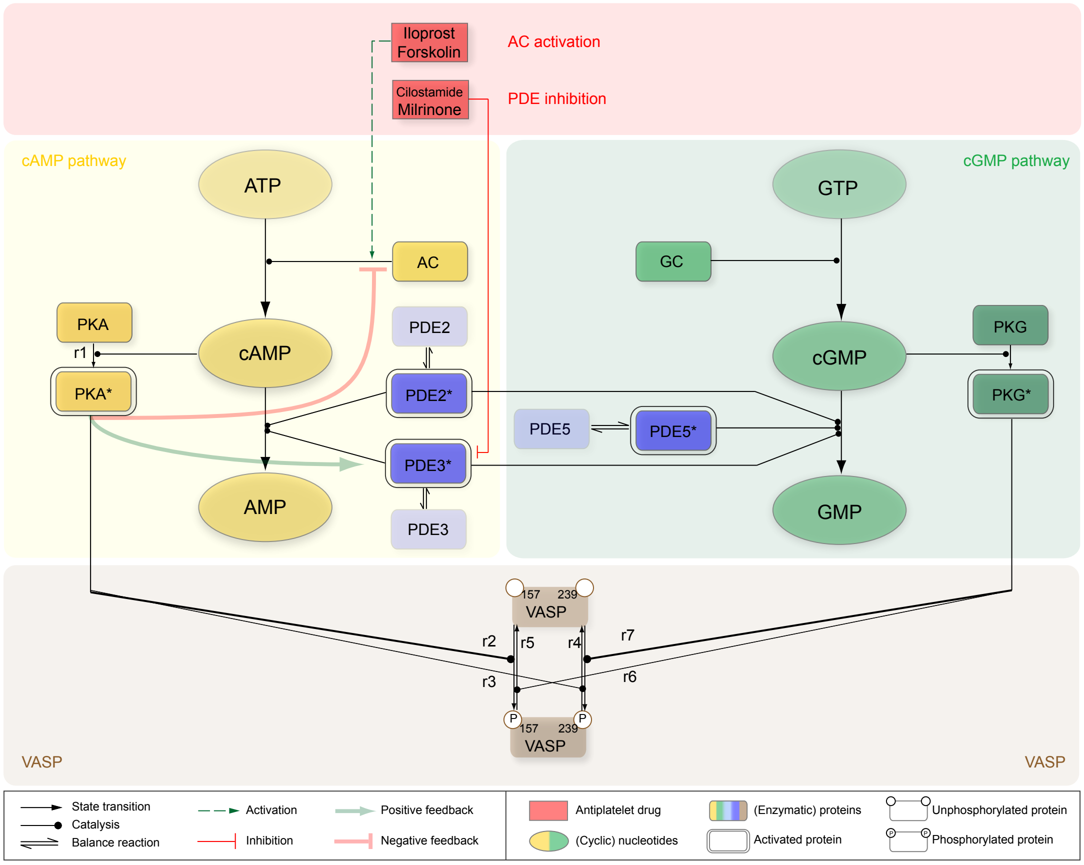

Fig S5.1 Reaction scheme of VASP phosphorylation - modeled reactions.

5.2 Variables and constants

Table S5.1 Set of variables and constants for the mathematical model of VASP activation.

**Dynamic variables**	**Values** and **fitting range**	**Remarks**
x_1: c(PKAα) inactive	Startvalue: 6.2 μM; fix	Catalytic subunit PKAα
x_2: c(VASP)	Startvalue: 25 μM; fix	Intracellular VASP concentration
x_3: c(VASP$_{Ser157}$) unphosphorylated	Startvalue: 25 μM;	Concentration of unphosphorylated VASP$_{Ser157}$
x_4: c(VASP$_{Ser239}$) unphosphorylated	Range: [0, 25] μM	Concentration of unphosphorylated VASP$_{Ser239}$
x_5: c(PKG) active	Startvalue: 1 μM; fix	Active c(PKG) due to basal cGMP level
x_6: c(PKAα) active	Simulated	
x_7: c(VASP$_{Ser157}$) phosphorylated<br>x_8: c(VASP$_{Ser239}$) phosphorylated	Simulated	Concentration of phosphorylated VASP$_{Ser157}$<br>Concentration of phosphorylated VASP$_{Ser239}$
**Constants**		
k_1: $kPKA\alpha_{act}$	Startvalue: 1<br>Range: $[0,1000]$	Activation of PKA through cAMP
k_2: $kVASP_{inact}$	Startvalue: 1<br>Range: $[0,1000000]$	Inactivation (dephosphorylation) of VASP
k_3: K_m VASP$_{Ser239}$	Startvalue: 10<br>Range: $[0,10000]$	PKA-specific
k_4: V_{max} VASP$_{Ser239}$	Startvalue: 1<br>Range: $[0,10000]$	
k_5: K_m VASP$_{Ser157}$	Startvalue: 0.1<br>Range: $[0,10000]$	
k_6: V_{max} VASP$_{Ser157}$	Startvalue: 1<br>Range: $[0,10000]$	
k_7: K_m VASP$_{Ser239}$	Startvalue: 10<br>Range: $[0,10000]$	PKG-specific
k_8: V_{max} VASP$_{Ser239}$	Startvalue: 10<br>Range: $[0,10000]$	
k_9: K_m VASP$_{Ser157}$	Startvalue: 10<br>Range: $[0,1000]$	
k_{10}: V_{max} VASP$_{Ser157}$	Startvalue: 10<br>Range: $[0,10000]$	
**Assignment rules**		
a_1: Ratio of VASP$_{Ser157}$ to VASP<br>a_2: Ratio of VASP$_{Ser239}$ to VASP		Experimental measurement
**Driving input**		
u_1: cAMP level<br>Time series within $[0,10]$ min		Due to Iloprost stimulation (2, 5, 10 nM)

Parameters $k_1 - k_{10}$ were fit to measured relations of phosphorylated VASP (Ser157, Ser239) to unphosphorylated VASP at several time points.

5.3 Rules

Defining the observed ratio between phosphorylated VASP and VASP in total:

$$\begin{aligned} &\text{Ratio of VASP}_{Ser157}\text{ to VASP:} \quad a_1 = \frac{x_7}{x_2}; \\ &\text{Ratio of VASP}_{Ser239}\text{ to VASP:} \quad a_2 = \frac{x_8}{x_2}; \end{aligned} \tag{5.1}$$

5.4 Reaction rate formalisms

$$\begin{aligned}
&\text{Activation of PKA via cAMP} && (r1): && v_1 = k_1 \cdot x_1 \cdot u_1; \\
&\text{Phosphorylation of VASP}_{Ser157}\text{ by PKA}\alpha && (r2): && v_2 = k_6 \cdot x_3 \cdot x_6/(k_5 + x_3); \\
&\text{Phosphorylation of VASP}_{Ser239}\text{ by PKA}\alpha && (r3): && v_3 = k_4 \cdot x_4 \cdot x_6/(k_3 + x_4); \\
&\text{Dephosphorylation of VASP}_{Ser239} && (r4): && v_4 = k_2 \cdot x_8; \\
&\text{Dephosphorylation of VASP}_{Ser157} && (r5): && v_5 = k_2 \cdot x_7; \\
&\text{Phosphorylation of VASP}_{Ser157}\text{ by PKG} && (r6): && v_6 = k_{10} \cdot x_3 \cdot x_5/(k_9 + x_3); \\
&\text{Phosphorylation of VASP}_{Ser239}\text{ by PKG} && (r7): && v_7 = k_8 \cdot x_4 \cdot x_5/(k_7 + x_4);
\end{aligned} \tag{5.2}$$

5.5 System of differential equations

Variables are defined in Table S5.1.

$$\begin{aligned}
\frac{dx_1}{dt} &= -v_1; \\
\frac{dx_2}{dt} &= 0.0; \\
\frac{dx_3}{dt} &= -v_2 + v_5 - v_6; \\
\frac{dx_4}{dt} &= -v_3 + v_4 - v_7; \\
\frac{dx_5}{dt} &= 0.0; \\
\frac{dx_6}{dt} &= +v_1; \\
\frac{dx_7}{dt} &= +v_2 - v_5 + v_6; \\
\frac{dx_8}{dt} &= +v_3 - v_4 + v_7;
\end{aligned} \tag{5.3}$$

5.6 Experimental data compared to model trajectories (VASP phosphorylation model 1)

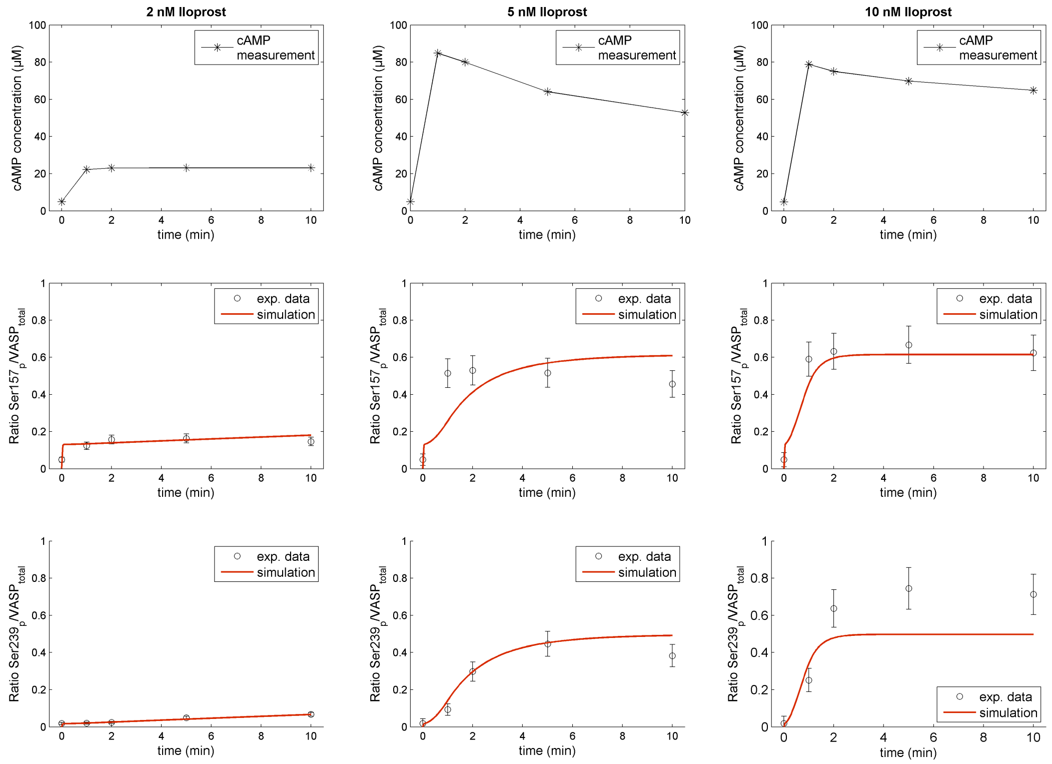

Fig S5.2 Fitted model trajectories and experimental measurements. Experimental data: Red circles are means of triplicate measurements (± SD; theoretically calculated). Curves: Calculated model trajectories obtained by fitting the model to the experimental data and simultaneously optimizing the parameters. First row: Elevated cAMP-level due to Iloprost stimulation (2 nM, 5 nM and 10 nM) over ten minutes. Second row: Ratio of VASP (phosphorylated at Ser157) to total VASP concentration at the three different stimulation levels. Third row: Ratio of VASP (phosphorylated at Ser239) to total VASP concentration.

6 VASP phosphorylation 2 - two distinct catalytic PKA subunits

Similarly to section 5, we here consider the modeling of VASP phosphorylation as downstream event but with two distinct variants of PKA (see Fig S6.1), one more active than its variant. Variables and constants of modeled reaction are listed in Table S6.1. Reaction rates, the resulting system of differential equations as well as time series data together with fitted model trajectories are given in sections 6.3-6.6. However, available data do not discriminate between the two VASP phosphorylation approaches (section 5 and 6). Hence, currently the more parsimonious model is to be preferred.

6.1 Reaction scheme

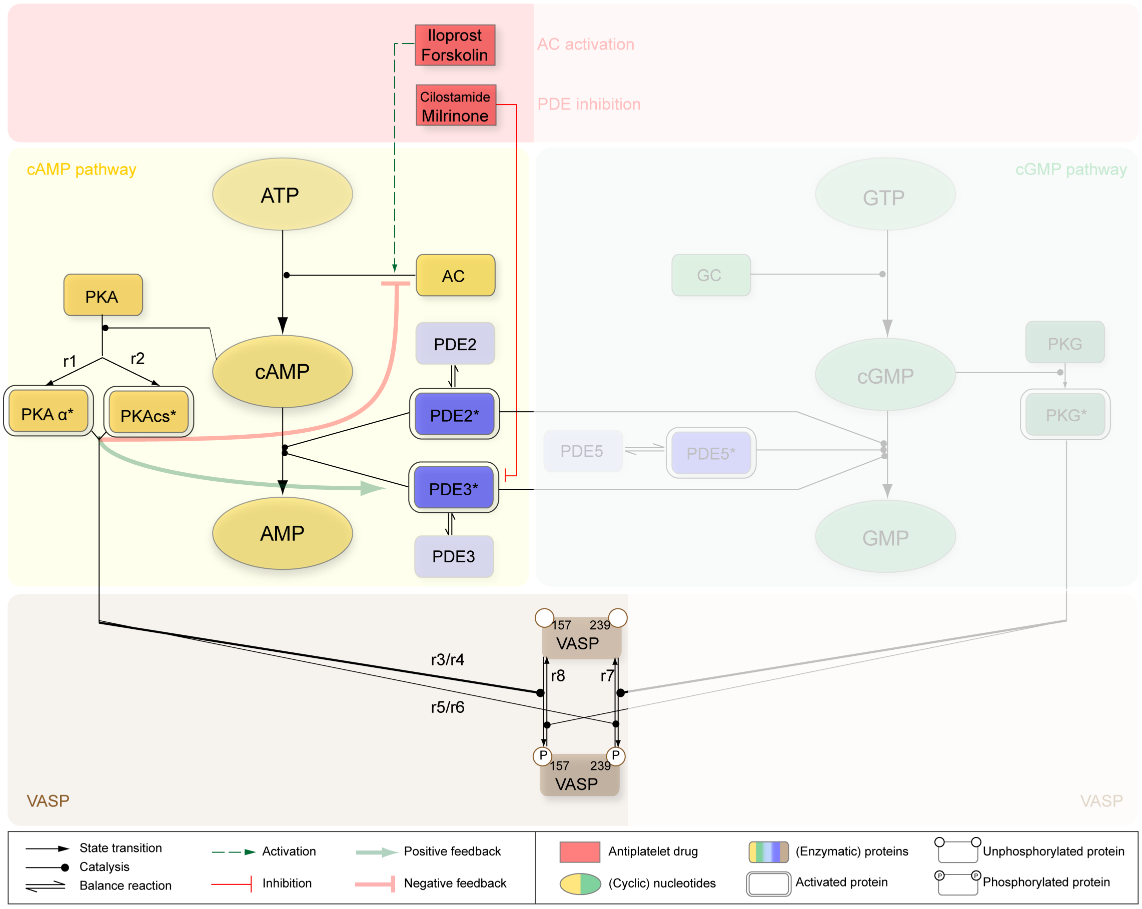

Fig S6.1 Reaction scheme of VASP phosphorylation - modeled reactions.

6.2 Variables and constants

Table S6.1 Set of variables and constants for the mathematical model of VASP activation.

**Dynamic variables**	**Values** and **fitting range**	**Remarks**
x_1: c(PKAα) inactive	Startvalue: 6.2 μM; fix	Catalytic subunit PKAα
x_2: c(PKAcs) inactive	Startvalue: 6.2 μM; fix	Distinct catalytic subunit of PKA
x_3: c(VASP)	Startvalue: 25 μM; fix	Intracellular VASP concentration
x_4: c(VASP$_{Ser157}$) unphosphorylated	Startvalue: 25 μM;	Concentration of unphosphorylated VASP$_{Ser157}$
x_5: c(VASP$_{Ser239}$) unphosphorylated	Range: [0, 25] μM	Concentration of unphosphorylated VASP$_{Ser239}$
x_6: c(PKAα) active	Simulated	Catalytic subunit PKAα
x_7: c(PKAcs) active		Catalytic subunit of PKA
x_8: c(VASP$_{Ser157}$) phosphorylated	Simulated	Concentration of phosphorylated VASP$_{Ser157}$
x_9: c(VASP$_{Ser239}$) phosphorylated		Concentration of phosphorylated VASP$_{Ser239}$
**Constants**		
k_1: $kPKA\alpha_{act}$	Startvalue: 1<br>Range: $[0, 1000]$	Activation of PKAα through cAMP
k_2: $kPKAcs_{act}$	Startvalue: 1<br>Range: $[0, 1000]$	Activation of PKAcs through cAMP
k_3: $kVASP_{inact}$	Startvalue: 1<br>Range: $[0, 1000000]$	Inactivation (dephosphorylation) of VASP
k_4: K_m VASP$_{Ser239}$	Startvalue: 10<br>Range: $[0, 1000000]$	PKAα-specific
k_5: V_{max} VASP$_{Ser239}$	Startvalue: 1<br>Range: $[0, 100000]$	
k_6: K_m VASP$_{Ser157}$	Startvalue: 0.1<br>Range: $[0, 100000]$	
k_7: V_{max} VASP$_{Ser157}$	Startvalue: 1<br>Range: $[0, 100000]$	
k_8: K_m VASP$_{Ser239}$	Startvalue: 1<br>Range: $[0, 1000000]$	PKAcs-specific
k_9: V_{max} VASP$_{Ser239}$	Startvalue: 1<br>Range: $[0, 1000000]$	
k_{10}: K_m VASP$_{Ser157}$	Startvalue: 0.1<br>Range: $[0, 100000]$	
k_{11}: V_{max} VASP$_{Ser157}$	Startvalue: 1<br>Range: $[0, 1000000]$	
**Assignment rules**		
a_1: Ratio of VASP$_{Ser157}$ to VASP		Experimental measurement
a_2: Ratio of VASP$_{Ser239}$ to VASP		
**Driving input**		
u_1: cAMP level<br>Time series within $[0, 10]$ min		Due to Iloprost stimulation (2, 5, 10 nM)

Parameters $k_1 - k_{11}$ were fit to measured relations of phosphorylated VASP (Ser157, Ser239) to unphosphorylated VASP at several time points.

6.3 Rules

Defining the observed ratio between phosphorylated VASP and VASP in total:

$$\begin{aligned} \text{Ratio of VASP}_{Ser157} \text{ to VASP:} \quad & a_1 = \frac{x_8}{x_3}; \\ \text{Ratio of VASP}_{Ser239} \text{ to VASP:} \quad & a_2 = \frac{x_9}{x_3}; \end{aligned} \tag{6.1}$$

6.4 Reaction rate formalisms

$$\begin{aligned} \text{Activation of PKA}\alpha \text{ via cAMP (r1)}: \quad & v_1 = k_1 \cdot x_1 \cdot u_1; \\ \text{Activation of PKA}cs \text{ via cAMP (r2)}: \quad & v_2 = k_2 \cdot x_2 \cdot u_1; \\ \text{Phosphorylation of VASP}_{Ser157} \text{ by PKA}\alpha \text{ (r3)}: \quad & v_3 = k_7 \cdot x_4 \cdot x_6/(k_6 + x_4); \\ \text{Phosphorylation of VASP}_{Ser157} \text{ by PKA}cs \text{ (r4)}: \quad & v_4 = k_{11} \cdot x_4 \cdot x_7/(k_{10} + x_4); \\ \text{Phosphorylation of VASP}_{Ser239} \text{ by PKA}\alpha \text{ (r5)}: \quad & v_5 = k_5 \cdot x_5 \cdot x_6/(k_4 + x_5); \\ \text{Phosphorylation of VASP}_{Ser239} \text{ by PKA}cs \text{ (r6)}: \quad & v_6 = k_9 \cdot x_5 \cdot x_7/(k_8 + x_5); \\ \text{Dephosphorylation of VASP}_{Ser239} \text{ (r7)}: \quad & v_7 = k_3 \cdot x_9; \\ \text{Dephosphorylation of VASP}_{Ser157} \text{ (r8)}: \quad & v_8 = k_3 \cdot x_8; \end{aligned} \tag{6.2}$$

6.5 System of differential equations

Variables are defined Table S6.1.

$$\begin{aligned} \frac{dx_1}{dt} &= -v_1; \\ \frac{dx_2}{dt} &= -v_2 \\ \frac{dx_3}{dt} &= 0.0; \\ \frac{dx_4}{dt} &= -v_3 - v_4 + v_7; \\ \frac{dx_5}{dt} &= -v_5 - v_6 + v_7; \\ \frac{dx_6}{dt} &= +v_1; \\ \frac{dx_7}{dt} &= +v_2 \\ \frac{dx_8}{dt} &= +v_3 + v_4 - v_8; \\ \frac{dx_9}{dt} &= +v_5 - v_6 - v_7; \end{aligned} \tag{6.3}$$

6.6 Experimental data compared to model trajectories (VASP phosphorylation model 2)

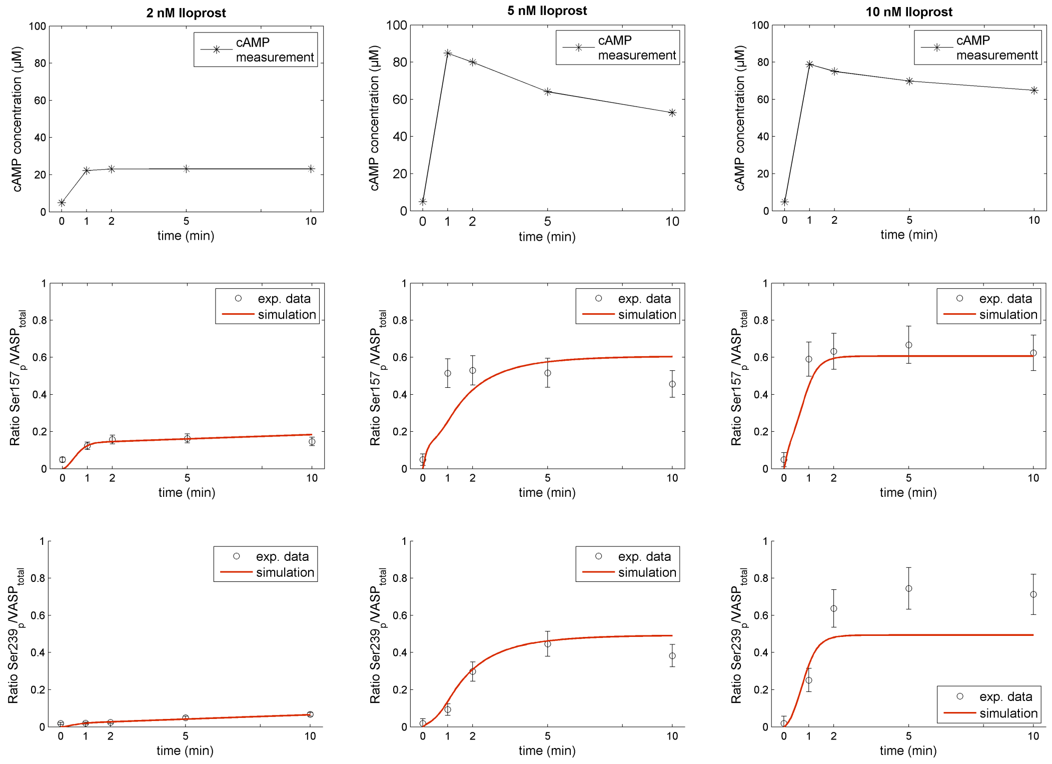

Fig S6.2 Fitted model trajectories and experimental measurements. Experimental data: Red circles are means of triplicate measurements (± SD; theoretically calculated). Curves: Calculated model trajectories obtained by fitting the model to the experimental data and simultaneously optimizing the parameters. First row: Elevated cAMP-level due to Iloprost stimulation (2 nM, 5 nM and 10 nM) over ten minutes. Second row: Ratio of VASP (phosphorylated at Ser157) to total VASP concentration at the three different stimulation levels. Third row: Ratio of VASP (phosphorylated at Ser239) to total VASP concentration.

7 Data-driven parameter fitting

The following table lists all paramter values fit to experimental data within the different modeling approaches (sections 2-6). All parameters describing PDE inhibition and AC activation, VASP phosphorylation (PKA/PKG, two distinct PKA sites) were obtained by fitting the corresponding models simultaneously to time series data.

Table S7.1 Fitted parameter values. Parameter symbols with respect to Table S3.1, Table S4.1, Table S5.1 and Table S6.1.

Parameter description	Parameter symbols	Value (best 50% of 1000 fits)	Value (best fit)
**PDE inhibition and AC activation**			
cAMP influx via AC in response to several application concentrations of Forskolin: (1 - 500 μM)	$AC_{\mathbf{Forskolin(500)}}$	595.60 ± 2.99 µmol/min	621.75 µmol/min
	$AC_{\mathbf{Forskolin(200)}}$	386.83 ± 2.01 µmol/min	403.01 µmol/min
	$AC_{\mathbf{Forskolin(100)}}$	85.84 ± 0.36 µmol/min	87.89 µmol/min
	$AC_{\mathbf{Forskolin(30)}}$	55.07 ± 0.30 µmol/min	55.60 µmol/min
	$AC_{\mathbf{Forskolin(10)}}$	29.20 ± 0.18 µmol/min	28.56 µmol/min
	$AC_{\mathbf{Forskolin(3)}}$	3.18 ± 0.14 µmol/min	2.10 µmol/min
	$AC_{\mathbf{Forskolin(1)}}$	6.32 ± 0.13 µmol/min	5.22 µmol/min
cAMP influx via AC in response to several application concentrations of Iloprost: (1 - 100 nM)	$AC_{\mathbf{Iloprost(100)}}$	116.23 ± 0.75 µmol/min	119.82 µmol/min
	$AC_{\mathbf{Iloprost(50)}}$	117.33 ± 0.75 µmol/min	120.93 µmol/min
	$AC_{\mathbf{Iloprost(10)}}$	68.21 ± 0.43 µmol/min	69.57 µmol/min
	$AC_{\mathbf{Iloprost(5)}}$	53.18 ± 0.35 µmol/min	53.89 µmol/min
	$AC_{\mathbf{Iloprost(1)}}$	4.63 ± 0.12 µmol/min	3.53 µmol/min
Inhibition of AC	k_{13}	0.86 ± 0.017 min^{-1}	0.69 min^{-1}
k_i Milrinone	k_{12_2}	0.16 ± 0.003 μM	0.14 μM
c(PDE2)	x_1	0.02 ± 0.003 µg/l	0.005 µg/l
c(PDE3)	x_2	1.78 ± 0.07 µg/l	1.70 µg/l
k_i Cilostamide	k_{12_1}	0.99 ± 0.003 μM	1 μM
Deactivation of PDE2	k_8	0.56 ± 0.018 min^{-1}	0.67 min^{-1}
Activation of PDE2	k_9	$0.0007 \pm 2.25 \cdot 10^{-5}$ min^{-1}	0.0008 min^{-1}
Deactivation of PDE3	k_{10}	0.02 ± 0.04 min^{-1}	0.95 min^{-1}
Activation of PDE3	k_{11}	$6.39 \cdot 10^{-5} \pm 0.0002$ min^{-1}	0.0046 min^{-1}
Basal influx of cAMP	k_6	8.93 ± 0.1 µmol/min	8.99 µmol/min
feedback regulation (cAMP → PDE3)	k_4	0.01 ± 0.01 µmol^{-1}	0.2 µmol^{-1}
**VASP** - model PKA and PKG			
kPKAα_{act} (2 nM)	k_1	$0.00028 \pm 1.64 \cdot 10^{-5}$ min^{-1}	0.00026 min^{-1}
kPKAα_{act} (5 nM)	k_1	0.008 ± 0.0015 min^{-1}	0.006 min^{-1}
kPKAα_{act} (10 nM)	k_1	0.04 ± 0.01 min^{-1}	0.03 min^{-1}
kVASP$_{\text{inact}}$	k_2	2.31 ± 0.53 min^{-1}	3.26 min^{-1}
K_mVASP$_{Ser239}$ (PKA$_\alpha$)	k_3	135.59 ± 61.32 μM	197.01 μM
V_{max}VASP$_{Ser239}$ (PKA$_\alpha$)	k_4	48.00 ± 22.95 µmol/min/mg	104.31 µmol/min/mg
K_mVASP$_{Ser157}$ (PKA$_\alpha$)	x_5	88.11 ± 38.07 μM	130.42 μM
V_{max}VASP$_{Ser157}$ (PKA$_\alpha$)	x_6	49.65 ± 22.05 µmol/min/mg	107.95 µmol/min/mg
K_mVASP$_{Ser239}$ (PKG)	k_7	12.36 ± 38.09 μM	2.05 μM
V_{max}VASP$_{Ser239}$ (PKG)	k_8	1.90 ± 2.45 µmol/min/mg	1.73 µmol/min/mg
K_mVASP$_{Ser157}$ (PKG)	x_9	158.28 ± 133.33 μM	214.44 μM
V_{max}VASP$_{Ser157}$ (PKG)	x_{10}	62.73 ± 47.46 µmol/min/mg	115.86 µmol/min/mg
**VASP** - model with two distinct catalytic PKA subunits			
kPKAα_{act} (2 nM)	k_1	63.54 ± 300.7 min^{-1}	0.18 min^{-1}
kPKAα_{act} (5 nM)	k_1	113.41 ± 390.59 min^{-1}	34.86 min^{-1}
kPKAα_{act} (10 nM)	k_1	930.42 ± 1670.26 min^{-1}	1.02 min^{-1}
kPKAcs$_{act}$ (2 nM)	k_2	$0.00033 \pm 3.99 \cdot 10^{-5}$ min^{-1}	0.00033 min^{-1}
kPKAcs$_{act}$ (5 nM)	k_2	0.0068 ± 0.001 min^{-1}	0.005 min^{-1}
kPKAcs$_{act}$ (10 nM)	k_2	0.02 ± 0.002 min^{-1}	0.02 min^{-1}
kVASP$_{\text{inact}}$	k_3	491.28 ± 168.90 min^{-1}	318.16 min^{-1}
K_mVASP$_{Ser239}$ (PKA$_\alpha$)	k_4	6.70 ± 11.49 μM	31.48 μM
V_{max}VASP$_{Ser239}$ (PKA$_\alpha$)	k_5	54.02 ± 20.16 µmol/min/mg	63.66 µmol/min/mg
K_mVASP$_{Ser157}$ (PKA$_\alpha$)	x_6	1.49 ± 4.58 μM	1.80 μM
V_{max}VASP$_{Ser157}$ (PKA$_\alpha$)	x_7	249.56 ± 85.36 µmol/min/mg	173.28 µmol/min/mg
K_mVASP$_{Ser239}$ (PKAcs)	k_8	0.76 ± 1.98 μM	0.01 μM
V_{max}VASP$_{Ser239}$ (PKAcs)	k_9	1041.23 ± 374.71 µmol/min/mg	676.98 µmol/min/mg
K_mVASP$_{Ser157}$ (PKAcs)	x_{10}	890.20 ± 445.65 μM	1805.42 μM
V_{max}VASP$_{Ser157}$ (PKAcs)	x_{11}	87429.4 ± 37233.7 µmol/min/mg	114700 µmol/min/mg

8 Parameters for drug combinations

There is a comprehensive list of drugs that affect cyclic nucleotide pathway signaling. In Table S8.1, several important drugs are given together with their specific parameters which serve as a basis for modeling and investigating effects of drug combinations.

Table S8.1 Drug specific parameter.

**Drug**	**Pathway effect**<br>(cAMP/cGMP)	**Drug specific parameter**	**Ref**	**Remark**<br>Model features tested
		Modeled		
Milrinone	PDE3 inhibition<br>(cAMP)	IC50: 56 ± 12 nM<br>IC50: 0.3 μM<br>IC50: 0.49 μM<br>IC50: 7.0 ± 0.9 μM<br>resulting in k_i values:<br>(0.05 - 0.3) μM<br>k_i: 0.55 μM<br>k_i: 0.66 μM<br>here: k_i: 0.15 μM	[11]<br>[12]<br>[13]<br>[14]<br><br>[15]<br>[16]<br>[17]<br>(k_{12_2}); Table S7.1[1]	single
Cilostamide		IC50: 70 ± 9 nM<br>IC50: 0.37 ± 0.005 μM<br>k_i: 1 μM	[11]<br>[14]<br>(k_{12_1}); Table S7.1[1]	
Iloprost	AC stimulation<br>(through GPCR)	k_{12_i}	section 4.3	
Forskolin	AC stimulation<br>(direct)			
		General/predictive		
EHNA<br><br>Oxindole<br>Bay-60 7550<br>PDP	PDE2 inhibition<br>(cAMP)	IC50: 1 μM<br>IC50: 0.8 - 4 μM<br>IC50: 40 nM<br>IC50: 4.7 nM<br>IC50: 0.6 nM	[18]<br>[19]<br>[20]<br>[21]<br>[22]	
Trequisin<br>Lixazinone<br>IBMX<br>Siguazodan	PDE3 inhibition<br>(cAMP)	IC50: 13 ± 2 nM<br>IC50: 22 ± 4 nM<br>IC50: 3950 ± 22 nM<br>IC50: 0.117 ± 0.029 μM	[11]<br>[11]<br>[11]<br>[11]	single
Zaprinast<br>Dipyridamole<br>Vardenafil<br>Sildenafil	PDE5 inhibition<br>(cGMP)	IC50: 0.76 μM<br>IC50: 0.9 μM<br>IC50: 0.16 ± 0.03 μM<br>IC50: 1.9 ± 0.4 μM	[18]<br>[18, 14]<br>[14]<br>[14]	
Milrinone-like agonists<br>Cilostamide-like agonists	PDE3 inhibition	Variation of k_{12_2}[2]<br>Variation of k_{12_1}[2]	see section 3.3, (r7)	
Iloprost-like agonists<br>Forskolin-like agonists	AC stimulation	Variation of k_{12_2}[2]<br>Variation of k_{12_1}[2]	see section 4.3, (r8)	
		Modeling drug combinations		
Milrinone/Iloprost<br>synergistic	PDE3 inhibition /<br>AC stimulation	Modified AC activation by<br>$k_{12} = 1.443 = \frac{cAMP}{k_{syn1}} > 1$	see section 4.3, (r8)	
Cilostamide/Iloprost<br>synergistic	PDE3 inhibition<br>AC stimulation	Modified AC activation<br>$k_{12} = 1.443 = \frac{cAMP}{k_{syn1}} > 1$	see section 4.3, (r8)	
Milrinone/AC inhibitor	PDE3 inhibition/<br>AC inhibition	k_3 (AC inhibitor (I) dependent)<br>here: $k_3 = 0.5$	*AC* influx (inhibited) = $k_6 - k_3 \cdot \log(c(I))$,<br>$k_6$: basal *AC* influx of cAMP	combined[3]
Forskolin/Milrinone<br>synergistic	PDE3 inhibition/<br>AC stimulation	Modified AC activation by<br>$k_{12} = 1.443 = \frac{cAMP}{k_{syn1}} > 1$	see section 4.3, (r8)	
Single drugs with<br>additive interaction		$k_{12} = 1$	see section 4.3, (r8)	
Single drugs with<br>antagonistic interaction	PDE3 inhibition/<br>PDE5 inhibition	$k_{12} = \frac{1}{1.443} < 1$	see section 4.3, (r8)<br>[23]	

[1] Estimated from experimental data
[2] Range according to pharmacological relevant differences in agonist strengths
[3] Several drug doses of each drug tested (see Figure 5, manuscript)

9 SBML Files

We created xml-files (CellDesigner 4.0.1) for the basal model (Additional file 3) and a more general model, comprising possible drug stimuli and downstream events of cyclic nucleotide signaling (Additional file 4). The latter can be adapted to various drug stimulation conditions by setting drug-specific constants (e.g. PDE specific inhibition constants k_i). Model topologies are depicted in Fig S9.1 (basal model) and Fig S9.2 (overall model).

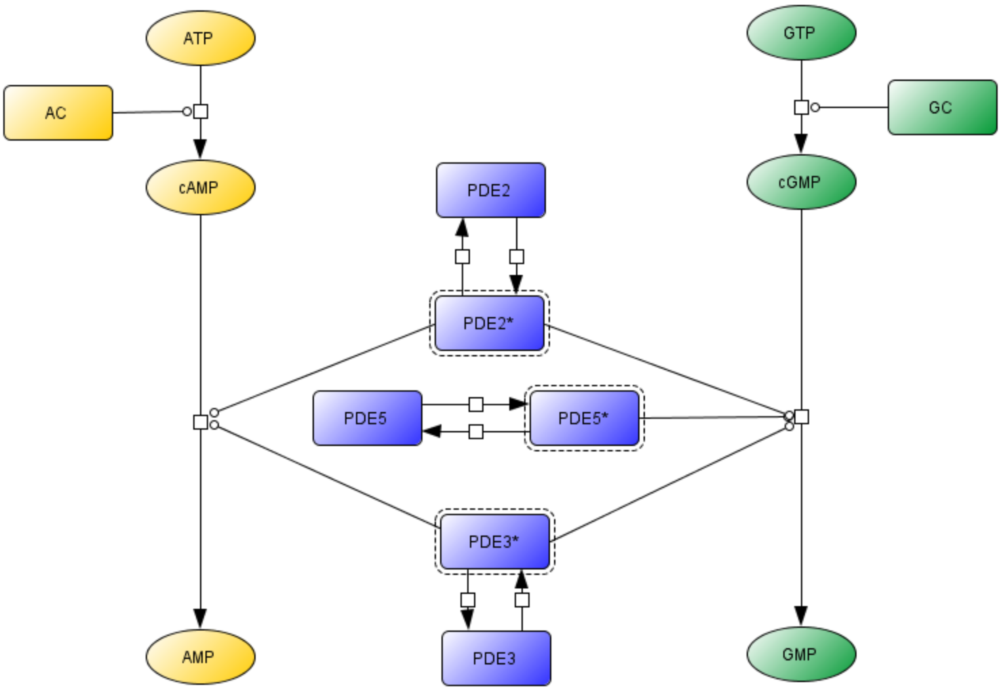

Fig S9.1 Topology of BasalModel.xml.

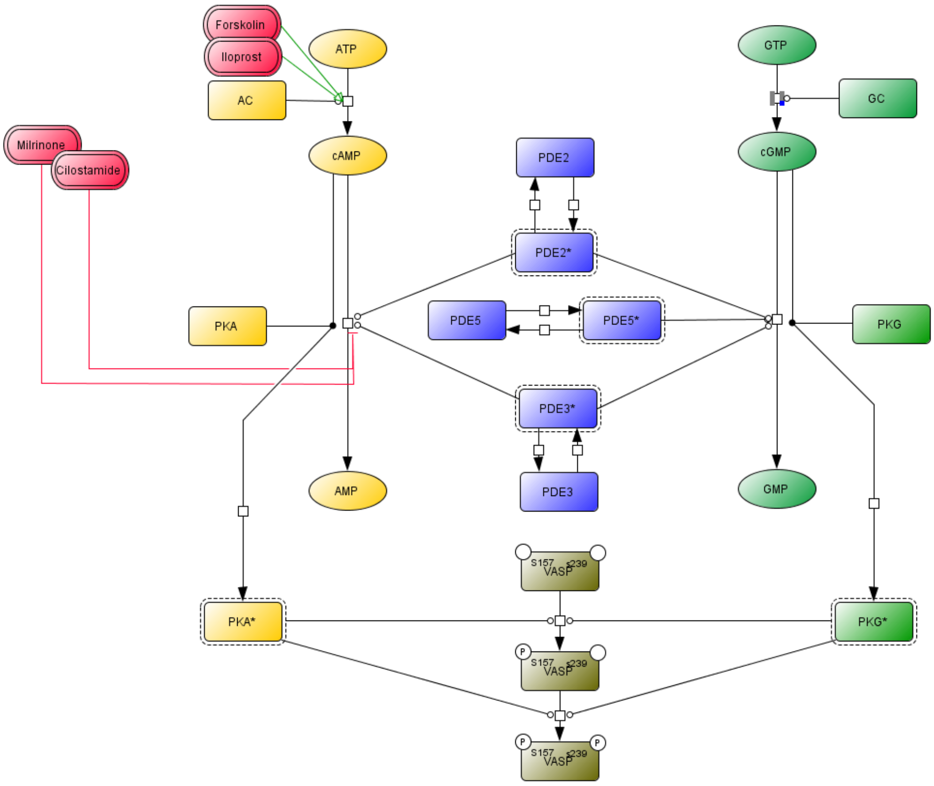

Fig S9.2 Topology of OverallModel.xml.

Part III
S3 Electron microscopy

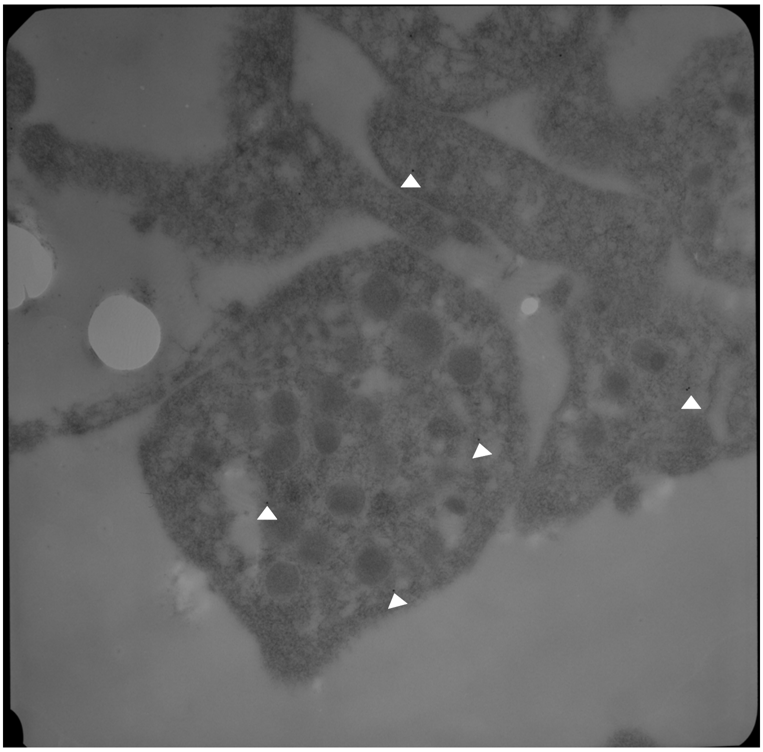

Fig S9.3 Electron microscopy (PDE5).

PDEs are only observed in low concentration but evenly distributed over the platelet (Fig S9.3; no membrane localization). Depicted are data for PDE5, where the antibodies gave best results (see arrows) supporting previous studies [24]. However, also micrographs for PDE2 and PDE3 gave similar results, but less prominent staining.

References

[1] Hines LM, Tabakoff B: **Platelet adenylyl cyclase activity: a biological marker for major depression and recent drug use**. *Biol Psychiatry* 2005, **58**(12):955–62. [WHO/ISBRA Study on State and Trait Markers of Alcohol Use and Dependence Investigators Journal Article Multicenter Study Research Support, N.I.H., Extramural Research Support, Non-U.S. Gov't United States].

[2] Parsian A, Todd RD, Cloninger CR, Hoffman PL, Ovchinnikova L, Ikeda H, Tabakoff B: **Platelet adenylyl cyclase activity in alcoholics and subtypes of alcoholics. WHO/ISBRA Study Clinical Centers**. *Alcohol Clin Exp Res* 1996, **20**(4):745–51. [Aa08028/aa/niaaa Aa09014/aa/niaaa Aa09515/aa/niaaa Journal Article Research Support, Non-U.S. Gov't Research Support, U.S. Gov't, P.H.S. United states].

[3] Juska A, Farndale RW: **Inhibition of human platelet adenylate cyclase activity by adrenaline, thrombin and collagen: analysis and reinterpretation of experimental data**. *Biochem J* 1999, **340 (Pt 1)**:245–53. [0264-6021 (Print) Comparative Study Journal Article].

[4] Eigenthaler M, Nolte C, Halbrugge M, Walter U: **Concentration and regulation of cyclic nucleotides, cyclic-nucleotide-dependent protein kinases and one of their major substrates in human platelets. Estimating the rate of cAMP-regulated and cGMP-regulated protein phosphorylation in intact cells**. *Eur J Biochem* 1992, **205**(2):471–81. [In Vitro Journal Article Research Support, Non-U.S. Gov't Germany].

[5] Schmidt K, Schrammel A, Koesling D, Mayer B: **Molecular mechanisms involved in the synergistic activation of soluble guanylyl cyclase by YC-1 and nitric oxide in endothelial cells**. *Mol Pharmacol* 2001, **59**(2):220–4. [0026-895X (Print) Journal Article Research Support, Non-U.S. Gov't].

[6] Butt E, Walter U: **Platelet phosphodiesterases**. *Handbook of experimental pharmacology* 1997, **126**:219–230.

[7] Bender AT, Beavo JA: **Cyclic nucleotide phosphodiesterases: molecular regulation to clinical use.** *Pharmacol Rev* 2006, **58**(3):488–520, [http://dx.doi.org/10.1124/pr.58.3.5].

[8] Dittrich M, Birschmann I, Pfrang J, Herterich S, Smolenski A, Walter U, Dandekar T: **Analysis of SAGE data in human platelets: features of the transcriptome in an anucleate cell**. *Thromb Haemost* 2006, **95**(4):643–51. [0340-6245 (Print) Journal Article Research Support, Non-U.S. Gov't].

[9] Smolenski A, Bachmann C, Reinhard K, Honig-Liedl P, Jarchau T, Hoschuetzky H, Walter U: **Analysis and regulation of vasodilator-stimulated phosphoprotein serine 239 phosphorylation in vitro and in intact cells using a phosphospecific monoclonal antibody**. *J Biol Chem* 1998, **273**(32):20029–35. [0021-9258 (Print) Journal Article Research Support, Non-U.S. Gov't].

[10] Butt E, Abel K, Krieger M, Palm D, Hoppe V, Hoppe J, Walter U: **cAMP- and cGMP-dependent protein kinase phosphorylation sites of the focal adhesion vasodilator-stimulated phosphoprotein (VASP) in vitro and in intact human platelets**. *J Biol Chem* 1994, **269**(20):14509–17. [0021-9258 (Print) Journal Article Research Support, Non-U.S. Gov't].

[11] Tang KM, Jang EK, Haslam RJ: **Photoaffinity labelling of cyclic GMP-inhibited phosphodiesterase (PDE III) in human and rat platelets and rat tissues: effects of phosphodiesterase inhibitors.** *Eur J Pharmacol* 1994, **268**:105–114.

[12] Bailey JM, Levy JH, Kikura M, Szlam F, Hug CC: **Pharmacokinetics of intravenous milrinone in patients undergoing cardiac surgery.** *Anesthesiology* 1994, **81**(3):616–622.

[13] Zhao H, Quilley J, Montrose DC, Rajagopalan S, Guan Q, Smith CJ: **Differential effects of phosphodiesterase PDE-3/PDE-4-specific inhibitors on vasoconstriction and cAMP-dependent vasorelaxation following balloon angioplasty.** *Am J Physiol Heart Circ Physiol* 2007, **292**(6):H2973–H2981, [http://dx.doi.org/10.1152/ajpheart.00419.2006].

[14] Herget S, Lohse MJ, Nikolaev VO: **Real-time monitoring of phosphodiesterase inhibition in intact cells.** *Cell Signal* 2008, **20**(8):1423–1431, [http://dx.doi.org/10.1016/j.cellsig.2008.03.011].

[15] Cer RZ, Mudunuri U, Stephens R, Lebeda FJ: **IC50-to-Ki: a web-based tool for converting IC50 to Ki values for inhibitors of enzyme activity and ligand binding.** *Nucleic Acids Res* 2009, **37**(Web Server issue):W441–W445, [http://dx.doi.org/10.1093/nar/gkp253].

[16] Floreani M, Fossa P, Gessi S, Mosti L, Borea PA, Dorigo P: **New milrinone analogues: in vitro study of structure-activity relationships for positive inotropic effect, antagonism towards endogenous adenosine, and inhibition of cardiac type III phosphodiesterase.** *Naunyn Schmiedebergs Arch Pharmacol* 2003, **367**(2):109–118, [http://dx.doi.org/10.1007/s00210-002-0675-2].

[17] Zhang W, Ke H, Colman RW: **Identification of interaction sites of cyclic nucleotide phosphodiesterase type 3A with milrinone and cilostazol using molecular modeling and site-directed mutagenesis.** *Mol Pharmacol* 2002, **62**(3):514–520.

[18] Soderling SH, Bayuga SJ, Beavo JA: **Identification and characterization of a novel family of cyclic nucleotide phosphodiesterases**. *J Biol Chem* 1998, **273**(25):15553–8. [0021-9258 (Print) Journal Article Research Support, U.S. Gov't, P.H.S.].

[19] Podzuweit T, Nennstiel P, Müller A: **Isozyme selective inhibition of cGMP-stimulated cyclic nucleotide phosphodiesterases by erythro-9-(2-hydroxy-3-nonyl) adenine.** *Cell Signal* 1995, **7**(7):733–738.

[20] Rivet-Bastide M, Vandecasteele G, Hatem S, Verde I, Bénardeau A, Mercadier JJ, Fischmeister R: **cGMP-stimulated cyclic nucleotide phosphodiesterase regulates the basal calcium current in human atrial myocytes.** *J Clin Invest* 1997, **99**(11):2710–2718, [http://dx.doi.org/10.1172/JCI119460].

[21] Boess FG, Hendrix M, van der Staay FJ, Erb C, Schreiber R, van Staveren W, de Vente J, Prickaerts J, Blokland A, Koenig G: **Inhibition of phosphodiesterase 2 increases neuronal cGMP, synaptic plasticity and memory performance.** *Neuropharmacology* 2004, **47**(7):1081–1092, [http://dx.doi.org/10.1016/j.neuropharm.2004.07.040].

[22] Seybold J, Thomas D, Witzenrath M, Boral S, Hocke AC, Bürger A, Hatzelmann A, Tenor H, Schudt C, Krüll M, Schütte H, Hippenstiel S, Suttorp N: **Tumor necrosis factor-alpha-dependent expression of phosphodiesterase 2: role in endothelial hyperpermeability.** *Blood* 2005, **105**(9):3569–3576, [http://dx.doi.org/10.1182/blood-2004-07-2729].

[23] Maurice DH: **Does sildenafil indirectly inhibit phosphodiesterase 3 in vascular smooth muscle?** *Hypertension* 2003, **41**(3):e2, [http://dx.doi.org/10.1161/01.HYP.0000054979.81019.0A].

[24] Wilson L, Elbatarny H, Crawley S, Bennett B, Maurice D: **Compartmentation and compartment-specific regulation of PDE5 by protein kinase G allows selective cGMP-mediated regulation of platelet functions**. *Proc Natl Acad Sci USA* 2008, **105**(36):13650–55.
