# Supplementary material for: Time-resolved in silico modeling of fine-tuned cAMP signaling in platelets: feedback loops, titrated phosphorylations and pharmacological modulation
Source: BMC Syst Biol. 2011 Oct 28;5:178. doi: 10.1186/1752-0509-5-178 (PMC3247139; doi:10.1186/1752-0509-5-178)
Supplement: Additional file 2 — Additional Results: Network sensitivity. Additional results: Sensitivity analysis and probing of the network sensitivity (permanent and transient model perturbations and pathway cross-linking). [file 1752-0509-5-178-S2.PDF]

Additional Results

1 Sensitivitiy Analysis

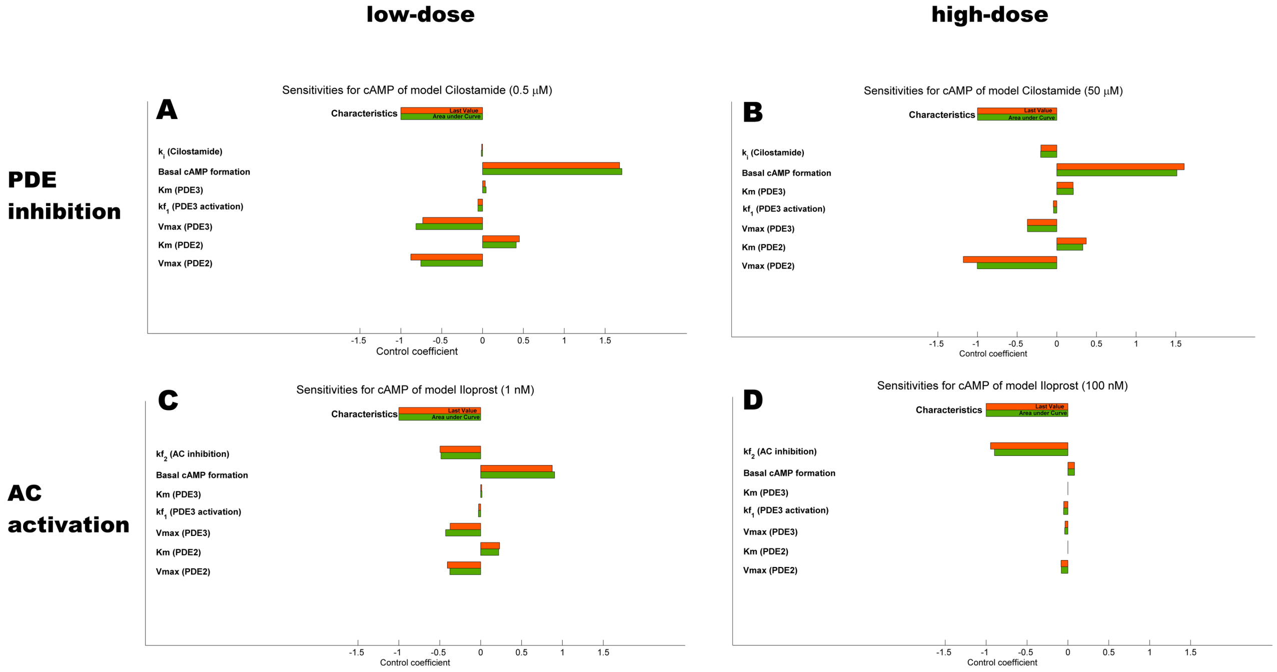

Fig S1.1 Detailed sensitivity analysis. Characteristics of the cAMP signal: *Area under Curve* (green) as a measure of the overall cAMP signal and *Last Value* (orange) denoting the magnitude of the cAMP plateau. PDE inhibition (**A**, **B**) and AC activation (**C**, **D**) were investigated separately in contrasting low-dose (**A**, **C**) with high-dose inhibition (**B**, **D**) of the platelet. Control coefficients represent the dependency of the characteristic value on a changed parameter value. Coefficients denote the increase of the signal property related to the increased parameter value. Calculating this ratio for all parameters shows similar profils for both signal characteristics (per drug concentration). The cAMP signal is mainly sensitive to the basal formation of cAMP (PDE inhibition) whereas the impact of drug- and PDE-specific constants augments with the increase of the inhibitory drug dose. Low-dose AC stimulation (**C**) result in similar characterstic profils, however for high-dose stimulation (**D**) via Iloprost (100 nM) the cAMP-dependent feedback constant (kf_2, feedback AC inhibition) outweighs the influence of the basal cAMP formation.

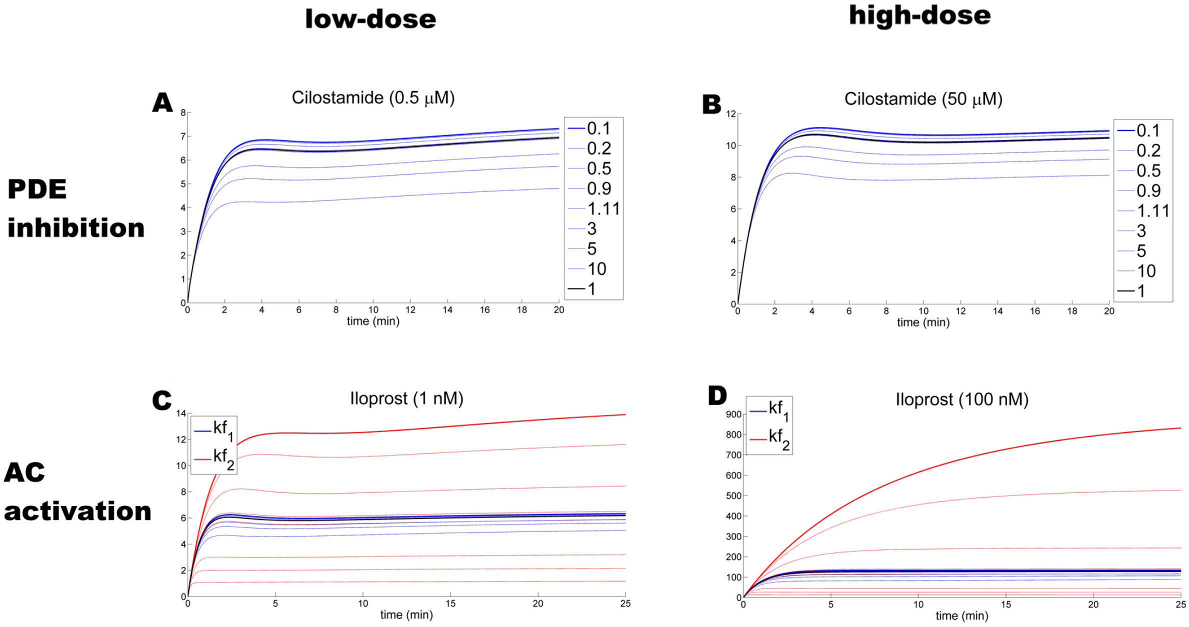

Fig S1.2 Sensitivity Analysis. Sensitivity of the feedback constants kf_1 (feedback activation of PDE3, blue) and kf_2 (feedback inhibition of AC, red, only modeled for AC stimulation conditions) on the cAMP signal. Constants were therefore multiplied by factors ranging from $0.1, 0.2, 0.5, 0.9, 1.11, 3$ and 5 to 10. Black trajectories denote the effect of the actual parameter value (factor 1). Starting from low to high-dose inhibition of PDE3, the variation of kf_1 resulted in cAMP signal spans ranging from 3 to 5 μM (**A**, **B**). Overall, the cAMP signal is highly sensitive to kf_2 (**C**, **D**).

2 Network sensitivity

2.1 Probing different time-scales of drug stimulation

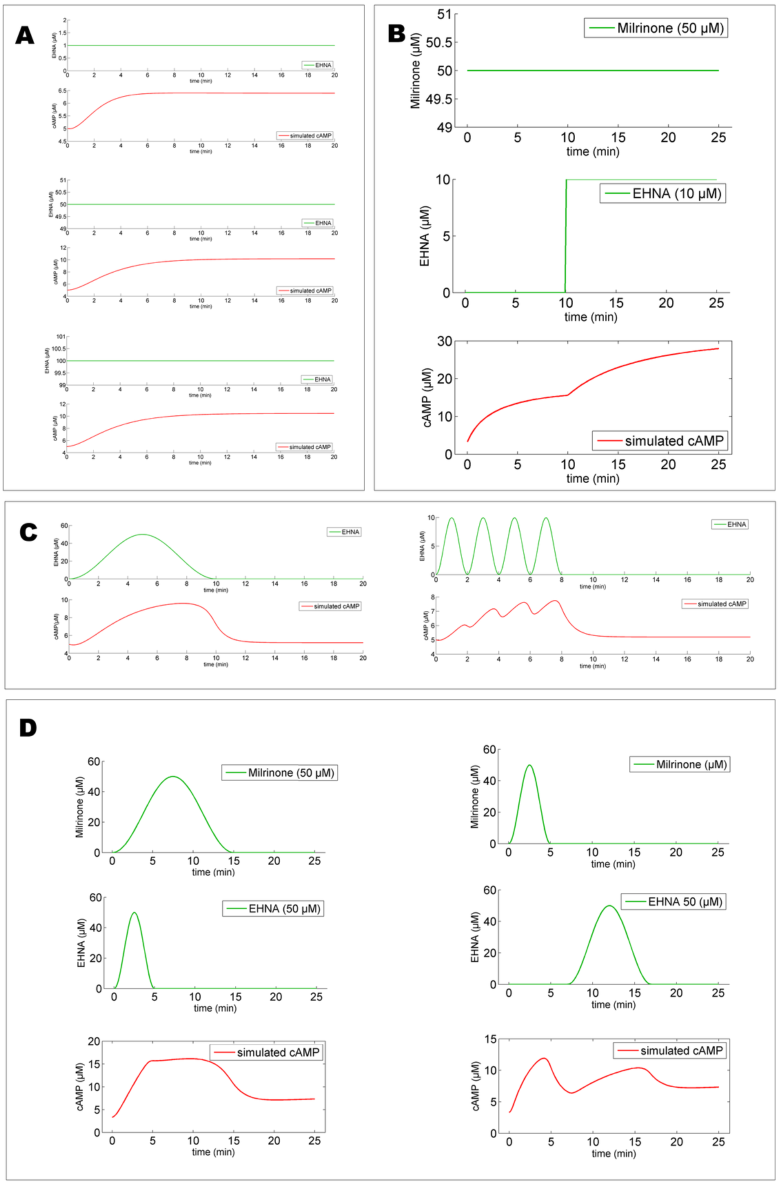

Fig S2.1 Probing different time-scales of drug stimulation. (**A**) Simulation of continuous inhibition of PDE2 (EHNA: specific inhibitor, assumed k_i-value: $0.7\mu M$). Model simulations result in increased cAMP plateau dependent on the inhibitor concentration. (**C**) The stimulation period of a single transient drug stimulus is mirrored in the predicted cAMP level, but dependent on the delay time between the stimulation pulses. (**B**) Successive continuous stimuli have additve effects on the cAMP level. This holds also for simultaneous and successive transient drug stimuli (**D**). Besides the drug doses, the signal magnitude (cAMP level) depends on the time interval between single drug pulses exemplified here for one single or two different PDE inhibitors (Milrinone and EHNA).

2.2 Transient prostacyclin stimulation

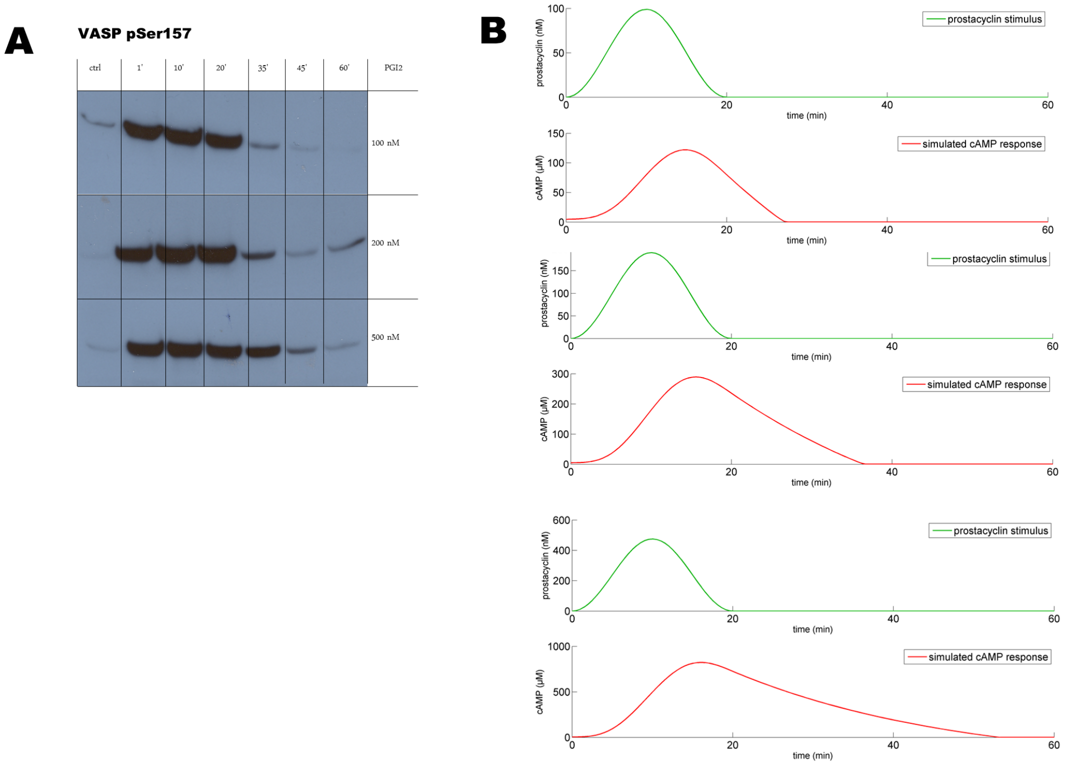

Fig S2.2 Transient prostacyclin stimuli. A Experimentally measured and **B** simulated responses to transient prostacyclin stimulations (doses of 100 nM, 200 nM and 500 nM). Both show transient effects on the cAMP level (panel **B**) and on the subsequent phosphorylation (VASP phosphorylation site Ser157, panel **A**) increasing with augmentation of the respective prostacyclin concentration. Model simulations: The established AC inhibition model (Iloprost, a prostacyclin-analogon) was perturbed by driving prostacyclin inputs (sine-pulse) of different amplitudes.

2.3 Theoretical cGMP → cAMP cross-talk

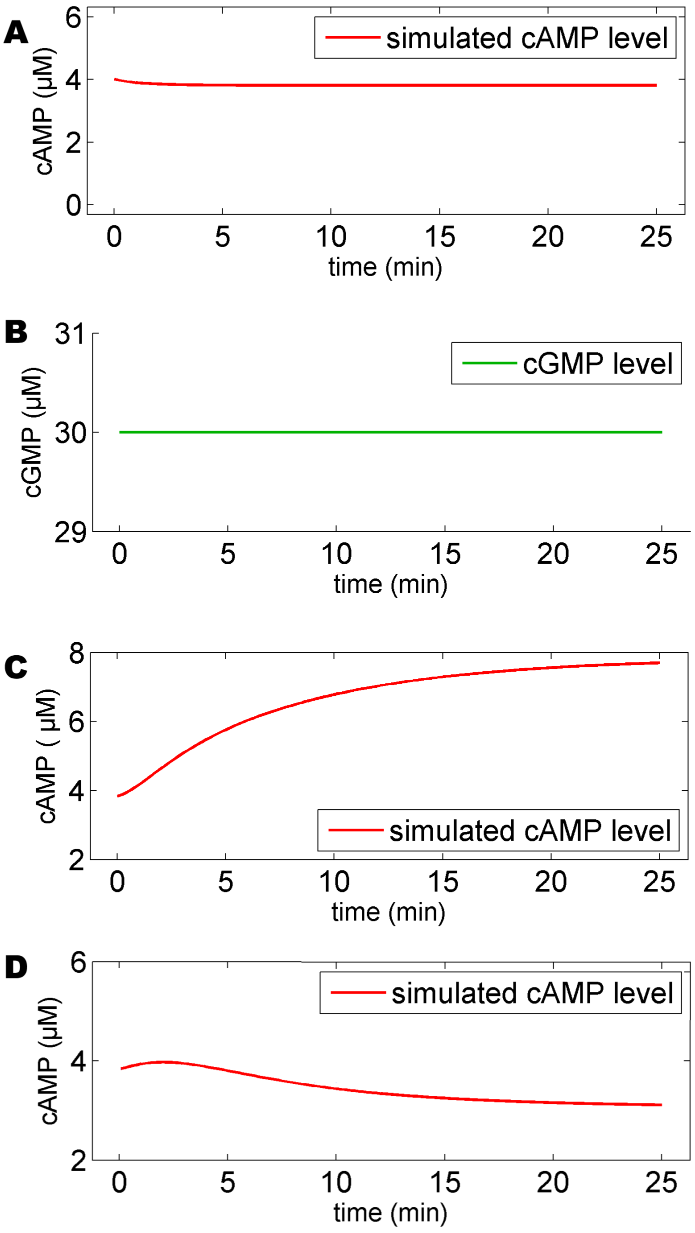

Fig S2.3 Theoretical pathway cross-talk. cGMP potentially decreases (via PDE2-stimulation) or enhances (via inhibition of PDE3) cAMP responses. To model this PDE cross-talk, we included two cGMP-dependent feedback constants (set to 0.1) in the basal model. Assuming an constant additional cGMP stimulus of 30 μM (**B**), the cAMP level does not remain at basal height (**A**) but rises (**C**). Thus, the model indicates a higher cGMP-dependency for PDE3 inhibition. However, increasing the feedback constant for cGMP-dependent PDE2 activation (2.5 fold) subsequently leads to an intensified cAMP dagradation (**D**).
